# Seasonal effects decouple SARS-CoV-2 haplotypes worldwide

**DOI:** 10.12688/f1000research.131522.1

**Published:** 2023-03-13

**Authors:** Tre Tomaszewski, Muhammad Asif Ali, Kelsey Caetano-Anollés, Gustavo Caetano-Anollés

**Affiliations:** 1 Department of Crop Sciences, University of Illinois at Urbana-Champaign, Urbana, Illinois, 61801, USA; 2 Callout Biotech, Albuquerque, New Mexico, 87112, USA; 3 C. R. Woese Institute for Genomic Biology, University of Illinois at Urbana-Champaign, Urbana, Illinois, 61801, USA

**Keywords:** AlphaFold2, epidemic calendar, membrane protein, mutation, protein interaction, protein structural prediction, proteome, seasonality, spike protein

## Abstract

**Background:** Variants of concern (VOCs) have been replacing each other during the still rampant COVID-19 pandemic. As a result, SARS-CoV-2 populations have evolved increasingly intricate constellations of mutations that often enhance transmissibility, disease severity, and other epidemiological characteristics. The origin and evolution of these constellations remain puzzling.

**Methods:** Here we study the evolution of VOCs at the proteome level by analyzing about 12 million genomic sequences retrieved from GISAID on July 23, 2022. A total 183,276 mutations were identified and filtered with a relevancy heuristic. The prevalence of haplotypes and free-standing mutations was then tracked monthly in various latitude corridors of the world.

**Results:** A chronology of 22 haplotypes defined three phases driven by protein flexibility-rigidity, environmental sensing, and immune escape. A network of haplotypes illustrated the recruitment and coalescence of mutations into major VOC constellations and seasonal effects of decoupling and loss. Protein interaction networks mediated by haplotypes predicted communications impacting the structure and function of proteins, showing the increasingly central role of molecular interactions involving the spike (S), nucleocapsid (N), and membrane (M) proteins. Haplotype markers either affected fusogenic regions while spreading along the sequence of the S-protein or clustered around binding domains. Modeling of protein structure with AlphaFold2 showed that VOC Omicron and one of its haplotypes were major contributors to the distortion of the M-protein endodomain, which behaves as a receptor of other structural proteins during virion assembly. Remarkably, VOC constellations acted cooperatively to balance the more extreme effects of individual haplotypes.

**Conclusions:** Our study uncovers seasonal patterns of emergence and diversification occurring amid a highly dynamic evolutionary landscape of bursts and waves. The mapping of genetically-linked mutations to structures that sense environmental change with powerful *ab initio* modeling tools demonstrates the potential of deep-learning for COVID-19 predictive intelligence and therapeutic intervention.

## Introduction

The COVID-19 pandemic continues unabated. There have been more than 750 million total cases and 7 million total deaths worldwide, with about half a million new cases being reported every day ( John Hopkins Coronavirus Resource Center). The makeup of SARS-CoV-2, the coronavirus responsible for the disease, is also changing at fast pace with the rise of numerous variants, some of which have impacted the success of vaccination and testing programs ( Williams & Burgers, 2021; Talenti *et al.*, 2022; McLean et al., 2022). Genomic changes arise from copying errors occurring during viral replication, the effects of host-induced editing (e.g. via host RNA deaminases), and recombination. Changes occur despite the SARS-CoV-2 genome being considered highly stable among positive-strand RNA viruses. This stability is endowed by its NSP14-mediated 3’-5’ exoribonuclease proofreading activities, which repair polymerase errors ( Ogando *et al.*, 2020) but also mediate extensive viral recombination ( Gribble et al., 2021). Within the context of evolving viral populations, the fate of mutations often depends on fitness (e.g. natural selection) or sampling (e.g. genetic drift, founder effects), by for example competitively enhancing viral replication, transmission rates, immune escape, or virulence. While too many deleterious mutations can push viral populations close to an ‘error threshold’ and the catastrophic possibility of extinction ( Domingo *et al.*, 2012), mutations that are not advantageous are generally eliminated. Instead, beneficial mutations often combine with each other to collectively increase the fitness of the viral ‘quasispecies’ ( Domingo *et al.*, 1978), structuring this dynamic ‘cloud’ of viral variants by linkage and collective effects on function and fitness ( Caetano-Anollés *et al.*, 2022).

Epidemiologically, viruses harboring either one mutation or unique constellations of mutations are generally referred to as ‘ *variants*’ ( Lauring & Hodcroft, 2021). These viral variants differ from *‘amino acid variants’* describing mutations that cause amino acid substitutions, insertions or deletions. Mutations that are statistically or experimentally linked to clinical or epidemiological criteria of significance (e.g., virus transmissibility, disease severity, or immune or vaccine escape) are considered ‘ *mutations of concern’* (MOCs). Variants with MOCs become an immediate priority for surveillance and response. This is particularly so when their prevalence increases worldwide. In particular, variants exhibiting constellations of MOCs are considered ‘ *variants of concern*’ (VOCs). The first VOCs of SARS-CoV-2 appeared in October 2020, few months after the first wave of the pandemic. Since their appearance, circulating VOCs have been replacing each other, generally increasing the number of accumulating mutations in each replacement round. The World Health Organization has named these VOCs with Greek letters according to their times of origin and is planning to use names of celestial constellations thereafter.

A constellation of mutations implies the existence of haplotypes (genetic signatures) and sets of mutations (markers, polymorphisms) that tend to be inherited together. Viral haplotypes represent mutations that are linked to each other, namely, that usually appear together in successful viral variants. This often results from beneficial intra-molecular or inter-molecular interactions operating at protein level. For example, the D614G mutation of the SARS-CoV-2 spike protein (S-protein), a substitution of an aspartic acid (D) by a glycine (G) at amino acid position 614 in the sequence of the S-protein, is part of a haplotype of four mutations including a P323L mutation in the NSP12 polymerase, a silent (F106F) mutation in the NSP3 papain-like protein, and a mutation in the 5’ untranslated region (UTR) of the genome. This first gene set was fixed in the worldwide viral population during the first wave (April 2020) of the COVID-19 pandemic ( Tomaszewski *et al.,* 2020). The haplotype is believed to have increased infectivity by enhancing the flexibility of the S-protein ( Voltz *et al.*, 2021). The S-protein is a trimer of highly glycosylated protomers, each harboring a N-terminal S _1_ subunit sequence with an N-terminal domain (NTD) and a receptor-binding domain (RBD), and a C-terminal S2 subunit holding a ‘fusion’ region and a ‘transmembrane’ region. The D614G mutation breaks a D614-T859 side chain hydrogen bond between the neighboring S _1_ and S _2_ subunits of pairs of protomers enhancing flexibility and subunit interactions ( Korber *et al.*, 2020). Cryo-electron microscopy (cryo-EM) analysis revealed that the mutation disrupted interprotomer contacts shifting the conformation towards an ACE-2-binding competent state necessary for membrane fusion with target cells ( Yurkovetskiy *et al.*, 2020). A subsequent conformational dynamics study showed that the mutation also affects the residues K854 and Y837 of the fusion peptide (FP) region contributing to linkage and/or allostery between the subunits ( Xu *et al.,* 2021). All VOCs have retained this initial haplotype but have added others as they appeared and were replaced, with each new constellation gathering a larger and more durable haplotype set by haplotype coalescence.

Since major VOCs such as Delta and Omicron have overtaken the entire global viral population, there is an implicit assumption that VOC mutant constellations represent relatively stable haplotypes. We recently tested this assumption by exploring the appearance and accumulation of VOC constellations in Australia as these were emerging throughout the COVID-19 pandemic ( Tomaszewski *et al.*, 2022). We chose Australia for two reasons. First, the country was able to control infection for the majority of the pandemic through effective disease mitigation strategies, including closing borders of states and country. This provided grounds to explore haplotype emergence in absence of significant effects from host migrations. Second, the Australian population is sparsely distributed and clumped into clearly defined urban areas along a significant latitude transect. This allowed testing effects of seasonal behavior on patterns of mutation accumulation since the beginning of the pandemic. The study revealed that 20 major haplotypes were associated with VOCs Alpha, Delta and Omicron in Australia and that there were significant recruitment episodes. Remarkably, core constellations showed significant decoupling patterns suggesting processes of emergence and significant and unanticipated seasonal patterns of diversification were at play in Australia. Decoupling manifested as latitude-imposed idiosyncratic patterns of accumulation within and between haplotypes.

Here we extend our exploration of haplotypes to the entire world. The GISAID initiative, sponsored by governments in partnership with public health and research institutions, has created a repository of genome data collected by extensive worldwide genome sequencing efforts ( Elbe & Buckland-Merrett, 2017; Shu & McCauley, 2017; Khare *et al.*, 2021). We mined levels of genetic variation unfolding in the evolving viral population using over 12 million genomic sequences retrieved from the GISAID repository on July 23, 2022. The construction of an ‘haplotype network’ that describes the worldwide viral population landscape throughout the COVID-19 pandemic confirmed significant decoupling patterns and increasing coalescence of haplotypes into larger haplotype groups. We also revealed seasonal patterns of emergence and diversification amid a highly dynamic viral evolutionary landscape. A protein interaction network mediated by haplotypes predicted molecular interactions, the effects of which could be tested with powerful *ab initio* structural modeling tools at atomic resolution. These results provide a unique window into our evolutionary understanding of a human pathogen of great significance.

## Methods

### Genomic analysis

The metadata for 12,070,698 SARS-CoV-2 genome sequences were downloaded on July 23, 2022, from the GISAID EpiCoV™ repository. Importantly, the metadata for each sequence contained a field listing all amino acid substitutions, referenced against the “hCoV-19/Wuhan/WIV04/2019” sequence (GISAID accession ID: EPI_ISL_402124, GenBank accession version: MN908947.3) ( Wu *et al.*, 2019). After extraction, data were filtered, limiting inclusion to sequences that were (a) obtained from a human host and (b) either labeled as “complete” *or* “high-coverage”. After filtering, metadata for 11,923,363 sequences remained, and these fields, along with other task-unnecessary metadata, were removed. See supplementary acknowledgements for the complete list of Accession IDs used.

To compare the prevalence of substitutions in climate zones, we split the “location” metadata field into the component parts of continent, country, region, and sub-region. The resulting country names were normalized for consistency and manually mapped to match a list of country coordinates assembled from a canonical dataset (available from DSPL, Google). The latitude was added as a field in the sequence metadata. These were then used to label a climate zone for each sequence. The scheme used five climate-zones: 30°S to 30°N latitudes as ‘Tropics’, 30°N to 60°N latitudes as ‘Northern Temperate’, 30°S to 60°S latitudes as ‘Southern Temperate’, and 60°N to 90°N latitudes as “Arctic”. No ‘Antarctic’ sequences corresponding to 60°S to 90°S latitudes were available. To identify the temporal dimension, we used monthly enumerations listing the month and year of sequence collection dates, starting on December 2019 (as index #0) and ending on July 23, 2022 (index #31).

Amino acid substitutions were isolated by collecting all variations occurring at least once within the lists present in the metadata “AA substitution” field for each sequence. They were labeled using accepted nomenclature ( den Dunnen & Antonarakis, 2000) from the Human Genome Variation Society. After aggregation, each of the 183,276 unique substitutions was split into component parts: (a) the protein name, (b) the reference amino acid, (c) the location of the amino acid in the protein, and (d) the amino acid substitution (deletion or insertion). The number of sequences per substitution over the entire period from December 2019 to July 2022 was counted. Since substitution groups containing low variance or spurious accumulation patterns were undesired for further analysis, they were filtered using a “relevancy” heuristic. We analyzed only substitutions with sequence counts greater or equal to one standard deviation over the mean of the entire sequence set.

Each sequence was indexed by the GISAID accession ID, climate zone, and monthly index, then (multi-)classified by amino acid substitution using a one-hot encoding (i.e., “1” identified the existence of the substitution in a sequence and “0” an absence). The incidence of each amino acid substitution for each climate-zone and month grouping was then calculated by summation across sequences and then divided by the total number of sequences within the climate zone-temporal period. For visualization, the monthly incidence of each climate zone was depicted in individual plots for each unique substitution using the Python library “matplotlib” v. 3.5.2 ( Caswell *et al.*, 2021; Hunter, 2007) and arranged and annotated using Adobe Illustrator v.25.2.3.

### Structural prediction analysis

Accelerated *ab initio* modeling of 3-dimensional atomic structures was conducted using the AlphaFold2 pipeline ( Jumper *et al.*, 2021) implemented locally in ColabFold without changes or modifications ( Mirdita *et al.*, 2022). The output of five ranked structural models was obtained following three neural network recycles (processing of predictions through models) that iteratively extracted co-evolutionary information in PDB70 structural templates and multiple sequence alignments (MSAs) for end-to-end training of the deep learning ‘evoformer’ and ‘structure’ multi-layered neural network modules. MSAs were built with fast and sensitive MMseqs2-based homology searches of UniRef100 and a database of environmental sequences. Accuracy was measured with the predicted local distance difference test (pLDDT) and the predicted aligned error (PAE). pLDDT provides a per-residue estimate of prediction confidence based on the LDDT-C _α_ metric ( Mariani *et al.*, 2013). The expected prediction reliability of a given region or molecule follows pLDDT ‘confidence bands’: >90, models with very high confidence; 90-70, models with confidence, showing good backbone predictions; 70-50, models with low confidence; and <50, models with very low confidence, generally showing ribbon-like structures. pLDDT <60 can be considered a reasonably strong predictor of intrinsic disorder. PAE measures confidence in the relative positions of pairs of residues, which evaluates the cohesiveness of structural modules (e.g., domains). Structural alignments and visualizations were carried out using Chimera ( Pettersen *et al.*, 2004). Reference (corresponding to M-EPI_ISL_402124) and variant structures were superimposed using the MatchMaker and MatchAlign tools to identify regions with structural divergences. Topological similarities of individual regions or entire molecules were evaluated with average template modeling scores ( TM-scores; Zhang & Skolnick, 2004) using USalign ( Zhang *et al.*, 2022a). M-protein predictions were benchmarked against two structural conformations recently acquired using cryo-EM ( Zhang *et al.*, 2022b). Besides TM-scores, GDT-TS scores were obtained using the LGA (local-global alignment) structure comparative analysis tool ( Zemla, 2003) with the AS2TS server, which CASP assessors routinely use to evaluate the accuracy of predicted structural models.

## Results

### Tracking the spread of SARS-CoV-2 variants

**Figure 1.**
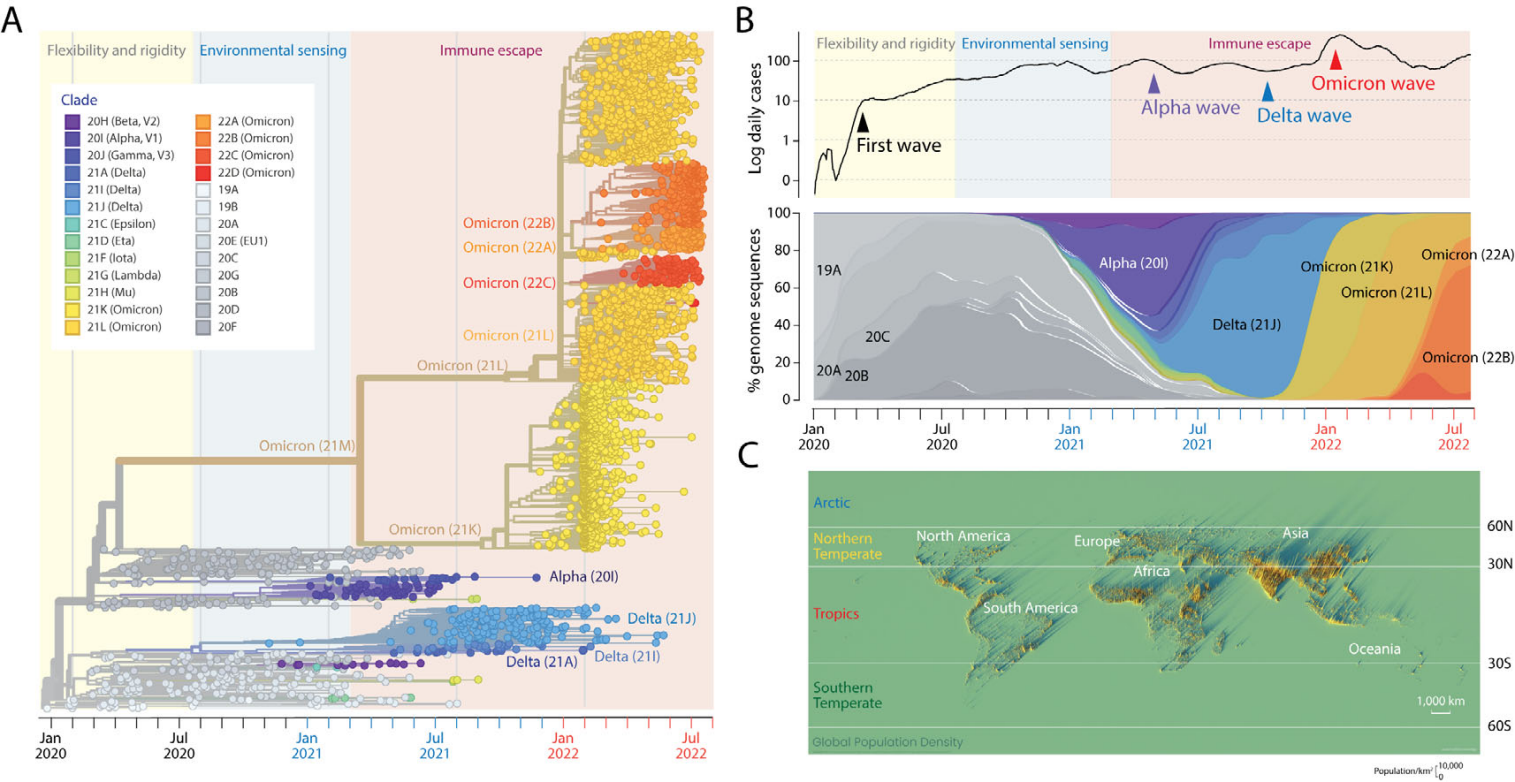
The mutational landscape of SARS-CoV-2 at the end of July 2022 and the spread of variants throughout the world during the pandemic. (A). A maximum likelihood phylogenetic tree describing the worldwide history of the SARS-CoV-2 genome. The timetree of 2,906 genomes randomly sampled between December 2019 and July 29, 2022 was obtained from Nextstrain. The tree unfolds the time of genome collection date from left to right. Its leaves (taxa indicated with circles) are colored according to the clade (group of taxa with a common evolutionary origin) and emerging variants of concern (VOCs) nomenclature. The origin of VOCs occurs when a clade originates along branches of the phylogeny. Note the early arrival of VOC Alpha, followed by VOC Delta and then VOC Omicron. The timeline of clades and VOCs shows three successive phases driven by proteome flexibility and rigidity, environmental sensing, and vaccine-driven immune escape, which are shaded in light yellow, blue, and salmon, respectively ( Caetano-Anollés *et al.*, 2022). (B). Plots showing the number of daily newly confirmed cases per million people (on a logarithmic scale and as 7-day rolling averages) and smooth percentages of genomes holding major VOCs since the beginning of the recorded COVID-19 pandemic. COVID-19 and genome data were retrieved from Johns Hopkins Univ., CSSE and GISAID, respectively. (C). Spike map showing a 3-dimensional representation of the population density of the world as a grid of vertical bars depicting the number of people per square kilometer of land area. Each spike represents the population in a grid of 2 km × 2 km. Light and shadow effects on the map highlight areas of high population density but also isolated population centers. Note the map shows no land. Instead it highlights locations where the 7.8 billion people of the world live (as of 2020). Labeled latitudes were used to split the world into four regions: Arctic, Northern Temperate, Tropics, and Southern Temperate, which are identified with colored letterings on the map and used to divide the genomic pool of the virus. The spike map is courtesy of Alasdair Rae, Automatic Knowledge Ltd., Sheffield, UK, reproduced with permission.

SARS-CoV-2 variants are organized around a master genomic sequence representing the virus that originated in the city of Wuhan in China. Many mutations have been added (some subtracted) to this master sequence since the beginning of the pandemic. These genomic changes can be traced by conducting a phylogenomic analysis of genomic sequences sampled throughout the disease timeline. Figure 1A shows a timetree reconstructed using the Nextstrain pipeline. It represents one out of 1,000 built from randomly sampled taxa. The trees have an implied time axis (measured in months) that allows dissecting viral spread, variant introductions, and rates of genomic change. Splits in the branches of the trees recovered using maximum likelihood optimality criteria define *clades*, groups of taxa with a common evolutionary origin (colored circles in the figure). They also define hypothetical ancestors embodying vectors of genomic sites that differ from the master sequence (the ancestor of all taxa) and change along branches as shared and derived phylogenetic features. These ancestors were recovered using character state reconstruction methods. As of July 2022, 26 Nextstrain clades defined by a year-letter nomenclature (beginning with clade 19A) and differing by at least two mutations from their parent major clades ( Hodcroft *et al.*, 2020) cataloged global viral diversity. Some splits in the tree describe the evolutionary appearance of major lineages. Figure 1B also tracks the accumulation of genomes corresponding to different VOCs, revealing how VOCs and their associated clades are being continuously replaced. For example, the VOC Omicron wave was represented by the clades 21K, 21L, 22A, 22B and 22C, which originated from a larger, more basal clade that at the very beginning of the pandemic gave rise to lineages that later led to VOC Alpha. Only three VOCs, Alpha, Delta and Omicron, became predominant worldwide at some point, completely replacing each other. VOC Alpha (clade 20I) appeared in the United Kingdom and was the first to expand quickly and globally probably due to increases in transmissibility and infection rates ( Davies *et al.*, 2021). VOC Delta (mostly 21J) was first identified in India in October 2020 but became predominant worldwide in June 2021. Finally, VOC Omicron was discovered in Botswana and South Africa in November 2021 before quickly spreading throughout the world in 2022.

### Dissecting the prevalence of amino acid variants along the timeline of the pandemic

**Figure 2.**
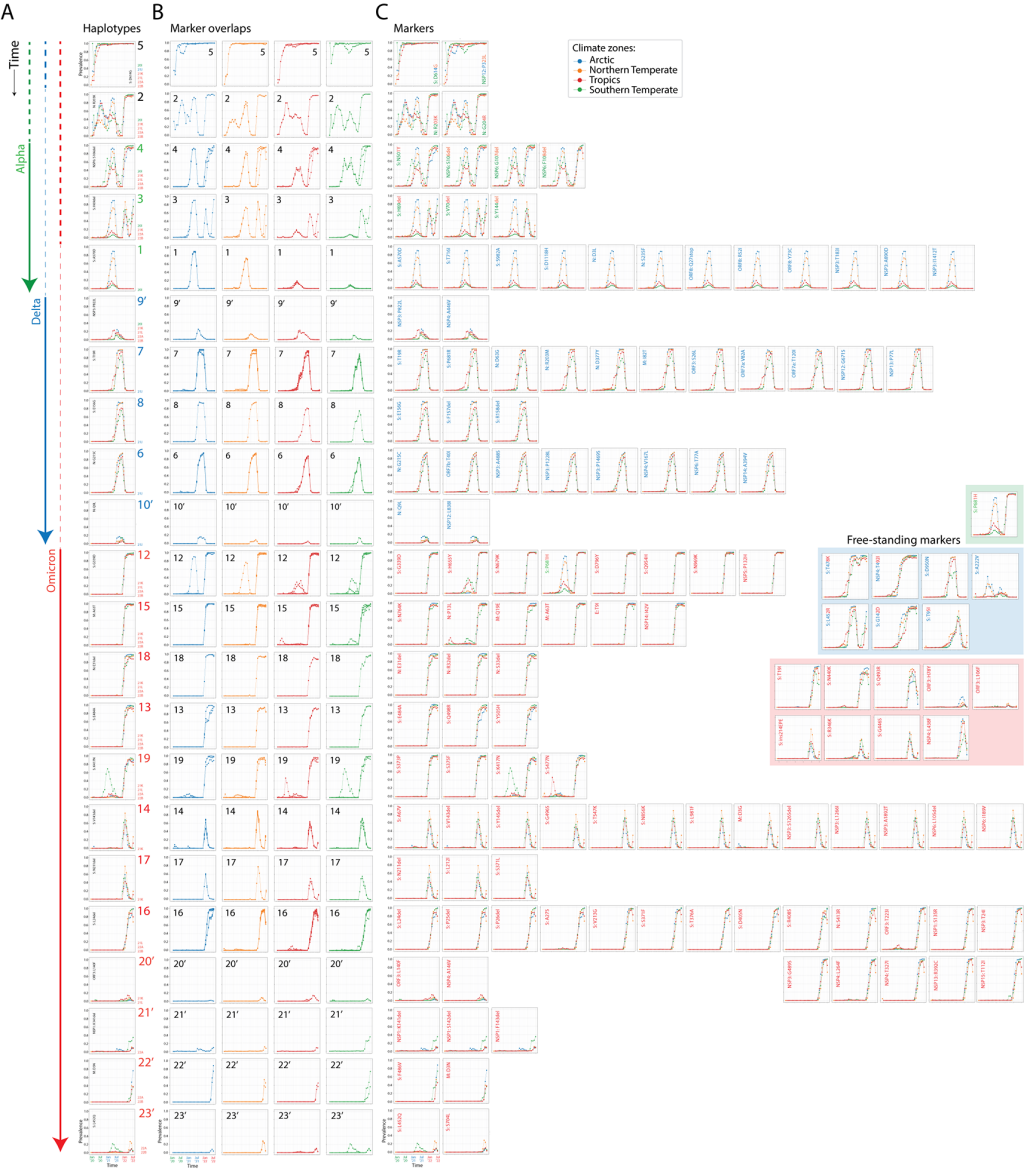
A chronology of SARS-CoV-2 haplotypes. (A) Accumulation plots illustrating haplotypes emerging along a timeline of the pandemic with labels colored according to VOCs they belong and time unfolding from top to bottom. The accumulation plot of a single mutant illustrates each haplotype. (B) Accumulation plot overlaps of all mutant markers of haplotypes describe haplotype decoupling for individual climatic zones. (C) Accumulation plots for mutants belonging to each haplotype are displayed from left to right. Mutant names are colored according to the VOCs they belong. The inset shows accumulation plots for free-standing markers.

To track the prevalence of individual mutations emerging throughout the pandemic, we analyzed 11,923,363 genomic sequences drawn from four climatic regions delimited by latitude worldwide ( Figure 1C), partitioning them into those acquired in each month of the pandemic. Regions included ‘Tropics’, ‘Northern Temperate’, ‘Southern Temperate’, and “Arctic” (latitude boundaries defined by 30°, 60° and 90° N and S); no ‘Antarctic’ sequences were available. Given background knowledge, latitude-delimited regions were expected to uncover seasonal effects in the global viral population ( Caetano-Anollés *et al.*, 2022). From these sequences, a total of 183,276 unique amino acid substitutions and insertion/deletions (variants) were identified and subsequently filtered with a “relevancy” heuristic that only kept those with a prevalence of greater or equal to one standard deviation over the mean of the entire sequence set. Note that the threshold, months, and regions are dynamic, and that the filtering criterion guarantees that we are not missing any significant mutations, especially those considered VOC markers. Accumulation plots of individual mutations corresponding to the major VOCs Alpha, Delta and Omicron ( Figure 2) were generated from the data and accumulation given as a prevalence. Prevalence ranged from 0 to 1, with 1 implying that 100% of genome sequences collected during an individual month contained that mutation. Collectively, accumulation plots describe the set of the most significant mutations affecting individual proteins of the viral proteomes in the different regions of the world and during each month of the pandemic.

### A chronology of haplotypes

**Figure 3.**
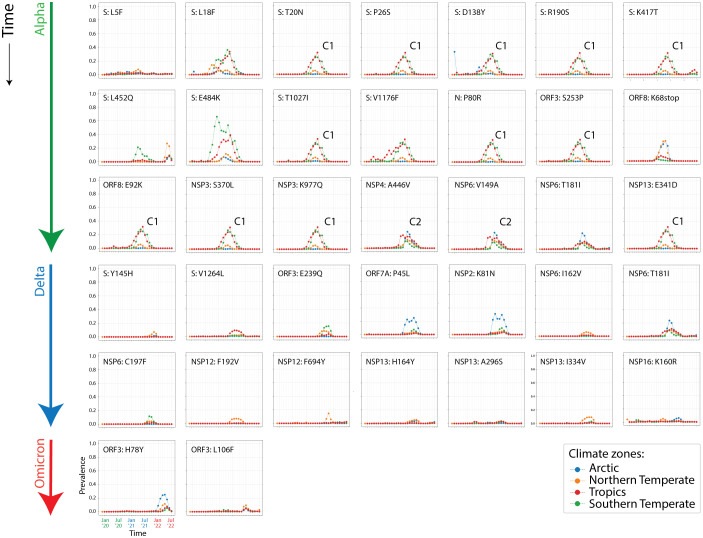
Other SARS-CoV-2 markers arising together with the VOC waves. Prevalence plots describing the accumulation of minor markers that failed to achieve large prevalence levels but were retained by the relevance heuristic. Note the existence of two cryptic haplotypes associated with the rise of VOC Alpha in Tropical and Southern Temperate corridors (C1 and C2).

Accumulation plots allowed us to define individual haplotypes, study their rise along a timeline, and evaluate their cohesiveness in the different climate zones of the world ( Figure 2). We defined haplotypes as sets of mutations harboring the same or very similar accumulation patterns during the lifetime of a VOC constellation ( Figure 1A). The cohesiveness of molecular and physiological interactions of a haplotype is however diminished when one or more mutations of a haplotype show distinct accumulation patterns. This ‘decoupling’ property can be uncovered by overlapping mutation accumulation plots for the four climate zones ( Figure 2B). Mutations were indexed according to their presence in the three widespread VOCs of the pandemic, Alpha, Delta and Omicron ( Figure 2C). We identified 22 haplotypes composed of 2–18 mutations affecting 1-8 proteins, one shared by the three VOCs and three shared by VOCs Alpha and Omicron. Two additional haplotypes belonged to minor VOCs listed in the accumulation plots of other markers identified with the relevancy heuristic ( Figure 3). Five free-standing mutations also unified the VOCs Omicron and Delta. Markers of VOC constellations confirmed the recruitment patterns we previously uncovered in Australia ( Tomaszewski *et al.*, 2022). Haplotype constitution was relatively conserved but also showed it was subject to the increasing trend of marker and haplotype accretion we highlighted worldwide ( Caetano-Anollés *et al.*, 2022) and in Australia ( Tomaszewski *et al.*, 2022). Indeed, we found five haplotypes holding 23 markers for the early VOC Alpha, six haplotypes holding 28 markers for VOC Delta, and 16 haplotypes holding 78 markers for the ongoing VOC Omicron. Thus, haplotypes and their associated markers have increased over time during our genomic sampling period. We note that six out of the 22 haplotypes we identified did not attain significant prevalence during the pandemic period analyzed; all labeled with prime symbols. For consistency, we named haplotypes following the nomenclature used in our Australian genomic study ( Tomaszewski *et al.*, 2022).

The first haplotypes appearing worldwide were *haplotype 5* and *haplotype* 2, in that order. *Haplotype 5* involves the well-known 4-mutation set mentioned above that includes the nonsynonymous D614G amino acid substitution of the S-protein and the P323L substitution of the NSP12 polymerase. The haplotype was first established in Europe during the first wave of the pandemic before spreading to other areas of the world. It is the most stable so far, is present in all VOCs, and is believed to increase COVID-19 infectivity ( Becerra-Flores & Cardozo, 2020; Korber *et al.*, 2020). Figure 2 shows that the emergence of the haplotype was noisy and decoupled across regions until November 2021, especially in the Arctic, Southern Temperate and Tropical regions of the world. *Haplotype 2* involves two mutations located in the intrinsically disordered serine/arginine-rich linker that separates the N-terminal and C-terminal RNA-binding domains of the nucleocapsid protein (N-protein), R203K and G204R ( Tomaszewski *et al*., 2020). Mutations were tightly coupled worldwide except for regions of the Northern Hemisphere during the first two quarters of 2021. Mutation prevalence was significant throughout the pandemic except for August-November 2021 (immediately before the rise of VOC Omicron), when mutation incidence decreased significantly worldwide. Prevalence patterns revealed two hemisphere-related waves, one centered around July 2020 that diminished prevalence in the Northern Temperate region, and another centered around April 2021 that fostered (decoupled) prevalence in Northern Temperate and Arctic regions. These patterns and the rise of the haplotype during the winter in the Southern Hemisphere suggest a seasonal effect. A similar conclusion was drawn from mutant accumulation plots in Australia ( Tomaszewski *et al.*, 2022). The haplotype was effectively recruited into VOC Omicron.

The mutant constellation of VOC Alpha introduced three additional haplotypes composed of 4-12 mutations affecting 1-4 proteins, *haplotypes 4*, *3*, and *1*, in that order ( Figure 2). The three haplotypes had larger incidences in the Northern Hemisphere, especially the last two. Their recruitment coincided with the second wave of *haplotype 2* we described above. *Haplotype 4* first appeared in the Southern Temperate region as a tightly linked set of four mutations, the N501Y mutant of the S-protein and three mutations in the autophagy-associated NSP6 protein that is linked to SARS. The haplotype extended relatively quickly worldwide, was tightly linked to markers of *haplotype 2* from July to November 2021 and was then coopted by VOC Omicron. *Haplotype 3* involved three mutations (deletions) affecting the NTD of the S-protein. Their accumulation followed that of *haplotype 4* but their incidence vanished from August to November 2021 before being coopted by VOC Omicron. Finally, *haplotype 1* involved 12 mutations affecting the S-protein, N-protein, the accessory ORF8 immune evasion protein, and the NSP3 papain-like proteinase scaffold. Markers belonging to each of all three haplotypes were tightly linked with each other, a pattern that differs from those of subsequent VOCs. As expected, however, prevalence of the three haplotypes across regions was low (below about 10% to 90%) following the low worldwide prevalence of VOC Alpha ( Figure 1B).

The mutant constellation of VOC Delta introduced five additional haplotypes harboring 2-11 mutations affecting 1-7 proteins—the S-protein, N-protein, membrane M-protein, ORF3, ORF7a, ORF7b, NSP3 protease, NSP4, NSP6, NSP12, and NSP14 exonuclease ( Figure 2). The widened diversity of haplotype proteins suggests VOC Delta significantly enhanced inter-molecular interactions. Except for *haplotypes 9’* and *10’*, haplotype prevalence reached 70% to 100% in all regions, with lower prevalence consistently evident in Tropical and Southern Temperate regions.

Finally, the core mutant constellation of VOC Omicron introduced an additional 12 haplotypes containing 2-18 mutants affecting 1-8 proteins—the S-protein, N-protein, M-protein, envelope protein (E-protein), ORF3, NSP1, NSP3, NSP4, NSP5, NSP6, NSP12, NSP13 and NSP14 ( Figure 2). *Haplotypes 13, 17, 19,* and *23’* altered sites exclusively present in the S-protein. *Haplotypes 12, 14,* and *16* also involved a significant number of S-protein markers. Overall, 37 out of 54 markers (69%) in these 12 haplotypes altered the S-protein, contrasting with only 14 S-protein markers out of 49 markers (29%) present in preceding haplotypes. This showed that the mutational landscape of the virus was becoming significantly biased towards the S-protein as the pandemic unfolded. Accumulation plots reveal most VOC Omicron haplotypes gained 100% prevalence in 3-4 months since the detection of the VOC in the Gauteng Province of South Africa in November 2021, much quicker than the six months it took for other VOCs to reach global or maximum prevalence levels. *Haplotypes 12* and *15* were the first to accumulate and reach solid 100% prevalence. *Haplotypes 13, 18, and 19* followed the same accumulation pattern but failed to reach global prevalence in Tropics and Southern Temperate regions. In turn, *haplotypes 14* and *17* struggled to reach 80% prevalence in January 2022 but then their incidence decreased in all regions, vanishing in Arctic and Southern Temperate regions. Conversely, *Haplotype 16* increased at a lower rate after a lag of a month only to reach 100% prevalence in the Arctic and Southern Temperate regions. The late appearing minor *haplotypes 20’-23’* showed vanishing tendencies or late increasing trends. Overall decoupling patterns suggest significant impact of latitude-related effects on the evolving genomic makeup of VOC Omicron.

Overlaps of accumulation plots for mutants in each haplotype revealed an increasingly significant decoupling of the VOCs Delta and Omicron haplotypes, with exceptions in *haplotypes 6, 8, 10’, 12, 18, 20’, 21’,* and *23’* ( Figure 2B) Decoupling regions were located in both the Northern and Southern Hemispheres. For example, *haplotype 15* was particularly decoupled in the Southern Temperate region while *haplotype 13* was particularly decoupled in the Arctic region.

### Emergence of haplotypes by recruitment of mutations in the viral population

Accumulation plots show that several haplotype markers appeared earlier in the pandemic than the haplotypes themselves ( Figure 2). As observed in Australia ( Tomaszewski *et al.*, 2022), the N-protein variant P13L of *haplotype 15*, which is associated with the N-terminal region of the nucleocapsid that is intrinsically disordered ( Tomaszewski *et al.*, 2020), appeared during the first wave of the pandemic between March and June of 2020 in the Tropics and Southern Hemisphere. The mutation was part of a pathway of mutational change involving protein flexibility/rigidity ( Tomaszewski *et al.*, 2020). The marker likely represents the oldest mutation of VOC Omicron other than those of *haplotypes 2* and *5.* Similarly, S-protein markers H655Y and P681H of *haplotype 12* appeared during the rise of VOC Alpha and S-protein markers K417N and S477N of *haplotype 19* appeared earlier in 2020. We also note that the A67V and V143del mutants of the S-protein and the D3G mutant of the M-protein of *haplotype 14* appeared before VOC delta in 2020. All of these patterns support the existence of significant recruitment operating during haplotype emergence (Tomaszewski *et al.*, 2021). This emergence is likely mediated by recombination, the process in which genomes of variants combine to form new variants during the replicative cycle of the virus.

**Figure 4.**
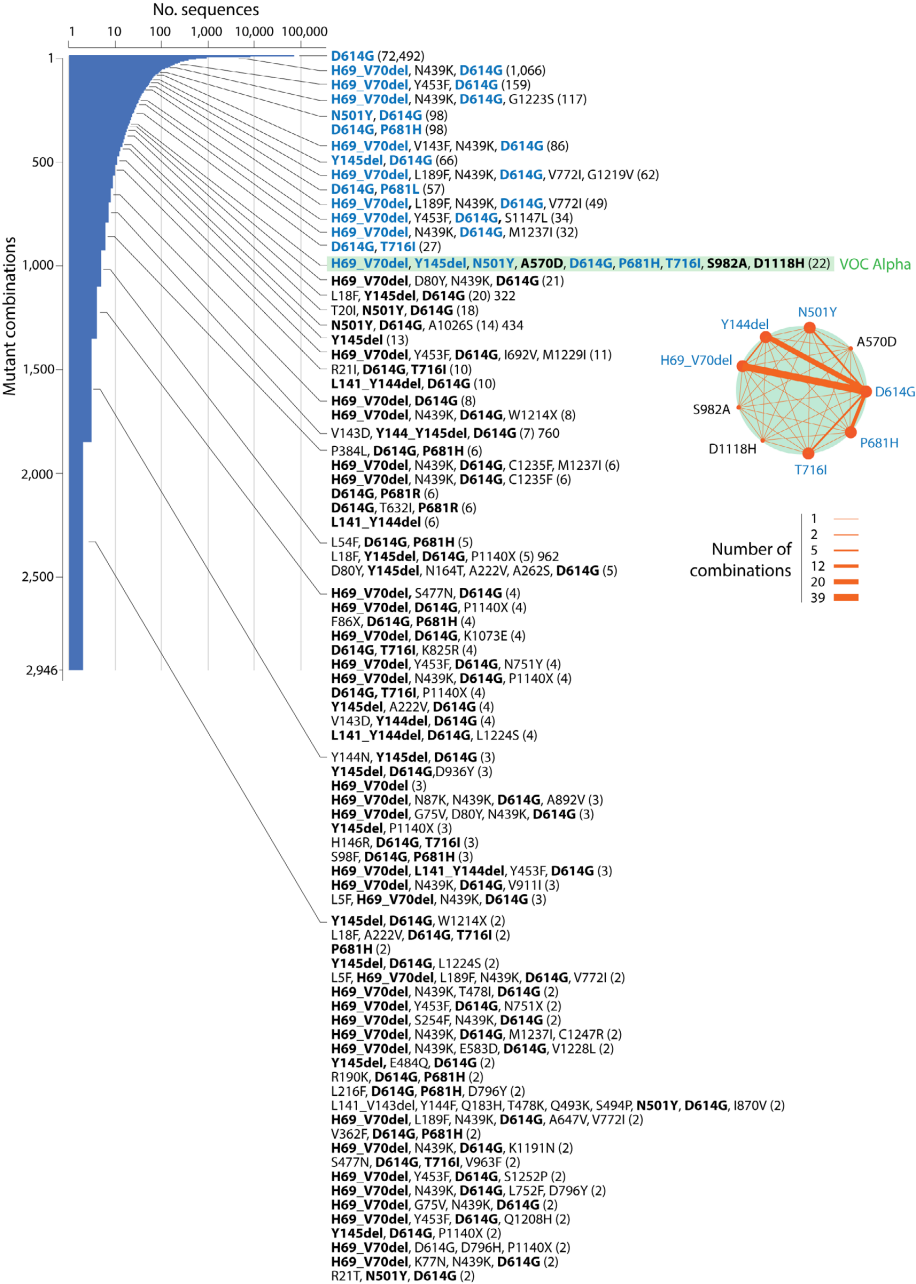
A frequency distribution plot describing the prevalence of S-protein mutant combinations appearing prior to VOC emergence during the first year of the COVID-19 pandemic. The plot is indexed with the names of 83 combinations harboring markers of the VOC Alpha constellation (in bold) and corresponding prevalence (number of sequences in parentheses). Note that VOC Alpha was reported a month after the sampling of the 137,605 S-protein sequences analyzed on November 14, 2020. Markers highlighted in blue have a higher prevalence than the 22 sequences of a single mutant combination harboring all markers of VOC Alpha (highlighted in green). They represent 67% of markers of that combination, offering ample opportunities for recombination. The inset shows a network of co-occurrence of markers of the VOC Alpha constellation. Nodes are mutations and links of the graph represent their co-occurrence. Data were retrieved from the Supplementary Tables in Showers *et al.* (2022).

The existence of a cloud of viral variants exploring a combinatorial landscape of mutations predicts that mutations and their combinations should precede the rise of VOC constellations. In a previous study we showed that VOC haplotypes recruited marker combinations already present in individual protein sequences before VOC emergence during late 2020 ( Caetano-Anollés *et al.*, 2022; Tomaszewski *et al.*, 2022). Here, we illustrate again the reuse of marker combinations in haplotypes by studying their presence in a dataset of 137,605 sequences of the S-protein retrieved worldwide on November 14, 2020 by Showers *et al.* (2022), exactly one month earlier than the announcement of VOC Alpha in the United Kingdom ( Public Health England, 2020). Counting the number of mutations in the S-protein sequences surveyed in the benchmarking study showed that most sequences harbored between 1-3 mutations with an average of 2.53 ± 0.94 (SE) mutations per sequence ( Table 1). The survey revealed that out of the 2,942 variant combinations identified, there were only 13 combinations with six mutations, one each with seven and eight mutations, and two with nine mutations in the data set. One of the two nine-mutant combinations (H69_V70del-Y145del-N501Y-A570D-D614G-P681H-T716I-S982A-D1118H) was present in 22 sequences and contained all VOC Alpha S-protein markers, including those of *haplotype 1* (A570D, T716I, S982A, D1118H), *haplotype 3* (H69del, V70del, Y145del), *haplotype 4* (N501Y) and *haplotype 5* (D614G), and the free-standing marker P681H, which collectively characterize the S-protein constellation of this viral variant. Note that Y144del of *haplotype 3* is indexed as Y145del in the dataset due to difficulties with identical adjacent amino acids in the alignment software ( Showers *et al.*, 2022). All 17 sequences contained at least one if not two to three of these markers, suggesting mutation increase in S-protein sequences was prerequisite for VOC emergence. Figure 4 maps the prevalence levels of the VOC Alpha mutations in the combinatorial landscape of 2020. The plot shows a strong bias in the prevalence of mutant combinations holding VOC Alpha markers, supporting a mechanism of VOC emergence via rearrangement rather than selective sweep in the viral population. Thus, going back to the first COVID-19 wave of 2020 reveals that VOC Alpha emerged by a combinatorial rearrangement of mutations already existent at different prevalence levels in the variant population.

**Table 1.** The combinatorial landscape of mutations arising in the S-protein during 2020.

Mutations/sequence *	Protein variants	Incidence (%)
1	297	9.8
2	1,315	44.7
3	907	30.8
4	329	11.2
5	77	2.6
6	13	0.4
7	1	0.03
8	1	0.03
9	2	0.06

* Average: 2.53±0.94 (SE) out of 2,942 variants. Regional deletions were counted as one mutation.

List of combinations containing six mutations with VOC Omicron haplotype markers labeled in bold and free-standing markers in italics: **H69_V70del**,L189F,N439K, **D614G**,V772I,G1219V (62 sequences) D80Y,N164T, *A222V*,A262S, **D614G**,P1140X (18 sequences) L5F, *A222V*,D574Y, **D614G**, **H655Y**,P1140X (11 sequences) D80Y, **Y145del**,N164T, *A222V*,A262S, **D614G** (5 sequences) D80Y, *T95I*,N164T, *A222V*,A262S, **D614G** (4 sequences) L5F, *A222V*,D574Y, **D614G**, **H655Y**,K1205N (4 sequences) D80Y,N164T, *A222V*,A262S, **D614G**,W1214X (3 sequences) D80Y,S98F,N164T, *A222V*,A262S, **D614G** (3 sequences) L5F, *A222V*,D574Y, **D614G**, **H655Y**,W1214X (3 sequences) L5F, **H69_V70del**,L189F,N439K, **D614G**,V772I (2 sequences) **H69_V70del**,L189F,N439K, **D614G**,A647V,V772I (2 sequences) L5F, *A222V*,N536S,D574Y, **D614G**, **H655Y** (2 sequences) D215V, *A222V*, **D614G**,P1140X,D1163Y,G1167V (2 sequences) List of combinations containing seven or more mutations with VOC Omicron haplotype markers labeled in bold and free-standing markers in italics: L141_V143del,Y144F,T478K, **E484K**,S494P, **D614G**,I870V(2 sequences) F65L,H66L,H66_A67insG,A67S,I68M,V70I,N439K, **D614G** (3 sequences) **H69_V70del**, **Y145del**, **N501Y**, **A570D**, **D614G**, **P681H**, **T716I**, **S982A**, **D1118H** (22 sequences) L141_V143del,Y144F,Q183H,T478K,Q493K,S494P, **N501Y**, **D614G**,I870V (2 sequences)

Remarkably, 58% of the VOC Omicron haplotype markers that appeared at the end of 2021 were already present in the accumulating 2,942 mutant combinations of November 2020, most of them at low prevalence levels ( Table 2). *Haplotypes 3, 4, 5, 13, 15*, and *16* had markers present in the 17 mutant combinations that had 6-9 mutations. Except for *haplotypes 15* and *22’*, all VOC Omicron haplotypes had markers representing them. Thus, haplotype primordia were already forming in the evolving viral population.

**Table 2.** Presence of S-protein mutations of VOC Omicron in 2020 prior to VOC emergence. The table lists the number of distinct mutant combinations harboring VOC Omicron markers out of the total 2,942 identified in a set of 137,605 sequences of the S-protein retrieved worldwide on November 14, 2020 ( Showers et al., 2022).

Mutations	Haplotype	Mutations/sequence								
		1	2	3	4	5	6	7	8	9
D614G	haplotype 5	1	1019	907	329	77	13	1	1	2
H69_V70del	haplotype 3	1	1	5	22	8	3	0	0	1
Y144del	haplotype 3	1	1	1	2	0	0	0	0	0
N501Y	haplotype 4	0	2	2	0	0	0	0	0	2
G339D	haplotype 12	0	0	0	3	0	0	0	0	0
H655Y	haplotype 12	1	2	8	1	1	4	0	0	0
N679K	haplotype 12	0	1	2	1	0	0	0	0	0
P681H	haplotype 12	1	1	6	1	0	0	0	0	1
D796Y	haplotype 12	0	1	1	1	1	0	0	0	0
Q954H	haplotype 12	0	0	0	0	0	0	0	0	0
N969K	haplotype 12	0	0	0	0	0	0	0	0	0
E484A	haplotype 13	0	0	1	0	0	0	0	0	0
Q493R	haplotype 13	0	0	0	0	0	0	0	0	0
Y505H	haplotype 13	0	0	0	0	0	0	0	0	0
A67V	haplotype 14	0	1	2	2	0	0	0	0	0
L141_V143del	haplotype 14	0	1	0	1	0	0	1	0	1
Y145del	haplotype 14	1	2	11	1	1	0	0	0	1
G496S	haplotype 14	0	0	0	0	0	0	0	0	0
T547K	haplotype 14	0	1	1	0	0	0	0	0	0
N856K	haplotype 14	1	0	0	0	0	0	0	0	0
L981F	haplotype 14	0	0	0	0	0	0	0	0	0
N764K	haplotype 15	0	0	0	0	0	0	0	0	0
L24del	haplotype 16	0	0	1	0	0	0	0	0	0
P25del	haplotype 16	0	0	0	0	0	0	0	0	0
P26del	haplotype 16	0	0	0	0	0	0	0	0	0
A27S	haplotype 16	1	1	5	1	0	0	0	0	0
V213G	haplotype 16	0	0	0	0	0	0	0	0	0
S371F	haplotype 16	0	0	0	0	0	0	0	0	0
S373P	haplotype 16	0	0	0	0	0	0	0	0	0
S375F	haplotype 16	0	0	0	0	0	0	0	0	0
T376A	haplotype 16	0	0	0	0	0	0	0	0	0
D405N	haplotype 16	0	0	0	0	0	0	0	0	0
R408S	haplotype 16	0	0	0	1	0	0	0	0	0
N211_L212del	haplotype 17	0	0	1	0	0	0	0	0	0
S371L	haplotype 17	0	0	0	0	0	0	0	0	0
K417N	haplotype 19	0	1	1	0	0	0	0	0	0
S477N	haplotype 19	0	1	119	20	1	0	0	0	0
F486V	haplotype 22'	0	0	0	0	0	0	0	0	0
L452Q	haplotype 23'	0	1	0	0	0	0	0	0	0
S704L	haplotype 23'	1	1	0	0	0	0	0	0	0

### A network view of emergence of haplotypes and mutant constellations

**Figure 5.**
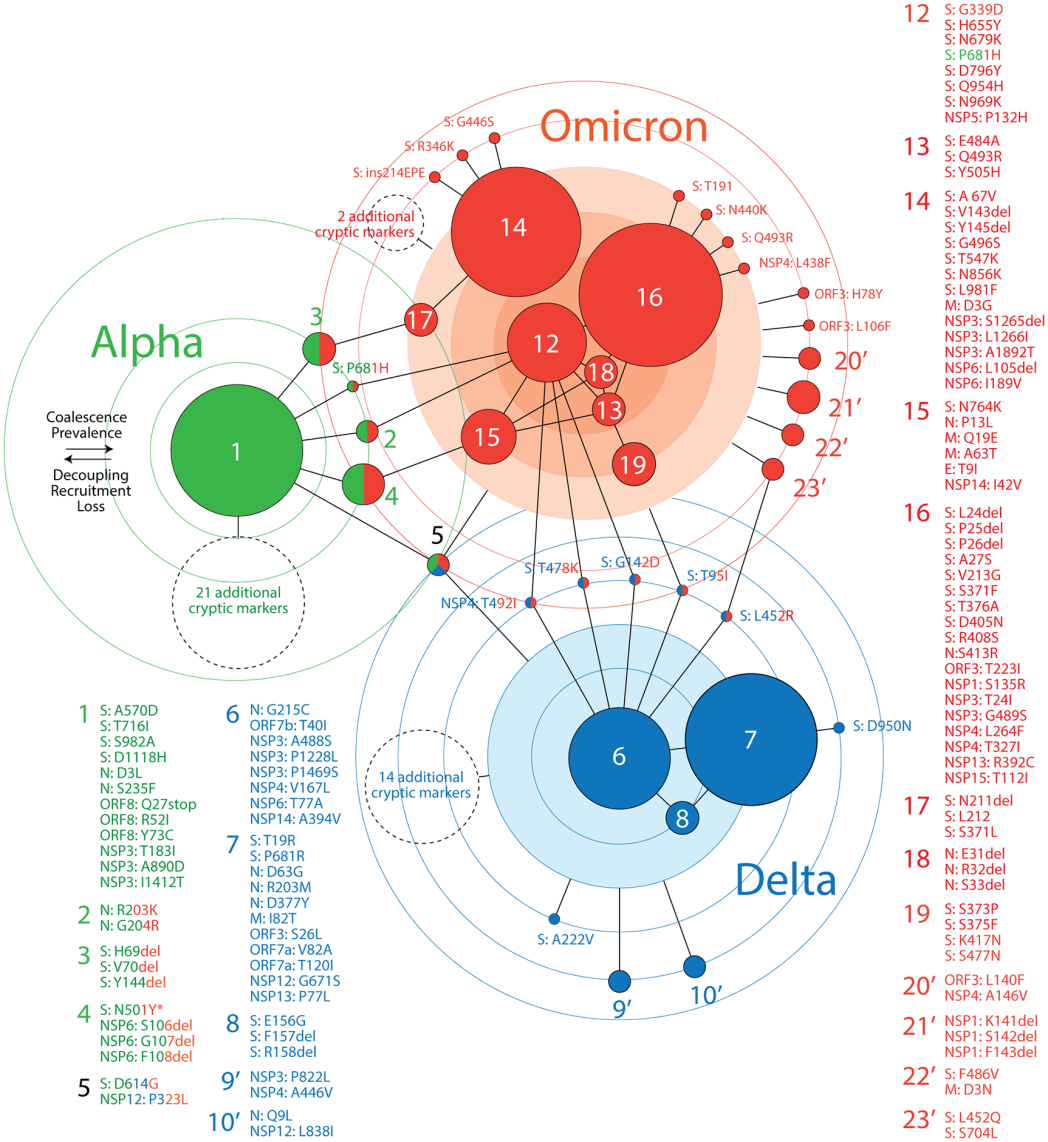
A network of haplotypes illustrating the worldwide emergence of major VOCs. Nodes and edges of the graph describe how haplotypes and free-standing mutations coalesce towards the inner-most circles of the major VOC constellations. Haplotype and mutant labels are colored according to their presence in VOCs worldwide. Cryptic markers are listed in Figure 3.

We constructed a ‘haplotype network’ describing the haplotype and mutant makeup of major VOCs ( Figure 5). The nodes of the graph are either haplotypes or free-standing mutations coalescing into VOC-specific mutant constellations. Node size is proportional to the number of haplotype markers. Edges describe common patterns of prevalence in accumulation plots. Circles portray levels of haplotype coalescence, that is, similarities in patterns of mutation accumulation of haplotype markers. Circles closer to the middle harbor mutants and haplotypes with prevalence patterns that are both similar and unique to each VOC constellation. Outer circles host mutants and haplotypes with patterns that are either more variable or shared by constellations of different VOCs. Mechanistically, haplotype coalescence unifies constellations pushing nodes to the middle of subgraphs and fostering hub behavior. In turn, seasonal decoupling, marker loss, and recruitment (sharing between constellations) push haplotypes and markers to their periphery. This frustrated interplay illustrates the dynamic mutational landscape of the virus.

The network in Figure 5 shows three clear subgraphs corresponding to the VOCs Alpha, Delta and Omicron being unified by the universally present *haplotype 5* and other shared haplotypes and free-standing markers. The network is structured by recruitment, coalescence, decoupling and loss:

(i) *Recruitment:* The constellation of VOC Omicron recruited three haplotypes ( *haplotypes 2, 3 and 4*) and one free-standing S-protein mutant (P681H) from VOC Alpha, and five free-standing mutants from VOC Delta. Together with *haplotype 5*, these recruitments impacted the S-protein (10 markers), N-protein (2 markers), NSP4 (1 marker), NSP6 (3 markers) and NSP12 (1 marker). Remarkably, VOC Alpha contributed almost half of its markers (11 out of 24 total) and 4 out of its 5 haplotypes to VOC Omicron, while VOC Delta contributed only a fifth of its markers (7 out of 35 total) and only 1 out of its 6 haplotypes to the makeup of the new VOC. Each episode of recruitment challenges the cohesive properties of the newly formed constellation because recruited haplotypes carry their own accumulation idiosyncrasies. For example, markers of *haplotype 5* were poorly linked during the rise of VOC Alpha but highly linked during the rise of VOC Omicron ( Figure 2). The reverse was true for markers of *haplotypes 3* and *4.*
(ii) *Coalescence:* We identified an apparent correlation between the time of origin of VOCs and haplotype coalescence and size. The 24 markers of the VOC Alpha constellation coalesced into five haplotypes, about half of which formed a single haplotype ( *haplotype 1*). Only one marker remained free-standing. The 35 markers of VOC Delta coalesced into six haplotypes, a third of which formed the largest haplotype (haplotype 7) of the set. Seven markers remained free-standing. In sharp contrast, the 92 markers of VOC Omicron organized into 16 haplotypes, about a fifth of which formed the largest haplotype (haplotype 16). 14 markers remained free-standing. The VOC Omicron constellation became stratified into a central core with at least three layers, one holding *haplotypes 12* and *18*, another holding *haplotype 16*, and a third holding *haplotypes 13*, *14*, *15*, *17,* and *19.* In addition, four peripheral haplotypes joined haplotypes recruited from VOC Alpha and a host of free-standing markers. Clearly, as VOCs replaced each other, markers increased in number but their constellations fragmented into increasingly more numerous haplotypes. The diversity of accumulation patterns also increased with the time of origin of VOC constellations. The typical single (sometimes noisy) single-peaked burst of mutation accumulation of VOC Alpha was replaced by multiple tightly overlapping bursts in VOC Delta and by both multiple-rate overlapping sigmoidal accumulations and distinct overlapping bursts in VOC Omicron ( Figure 2).
(iii) *Decoupling:* Markers tightly linked to each other in a haplotype are expected to show minimum differences in accumulation unless the molecular interactions or physiologies they mediate are affected by environmental, behavioral or physiological drivers. In our study, the decoupling effects of latitude on mutation prevalence tested the cohesiveness of individual haplotypes in VOC constellations. The monolithic behavior of *haplotype 1* of VOC Alpha, *haplotypes 6* and *8* of VOC Delta, and *haplotypes 12* and *18* of VOC Omicron placed them at the core of their respective constellations, while more variable haplotypes were more peripheral (e.g. *haplotypes 4*, *7* and *16*). Recall that decoupling manifests as idiosyncratic patterns of accumulation within and between haplotypes. VOC Alpha constellations hosted two types of haplotypes, one widely prevalent and exhibiting highly consistent accumulation patterns in the Northern Hemisphere ( *haplotypes 1* and *3*), the other quite noisy. Similarly, the steady gain of the high prevalence of most haplotypes of VOC Omicron was countered by the ‘burst’ behavior of *haplotypes 14* and *17* driven by the replacement of the initial 21K clade of VOC Omicron ( Figure 1B).
(iv) *Loss:* In contrast with the steady and highly prevalent levels of *haplotype 5*, the early but steady *haplotypes 2, 3*, and *4* showed episodes of gain and loss along the timeline of the pandemic ( Figure 2). The haplotypes appearing later were lost once VOCs started replacing each other. Free-standing markers were also lost, except for G142D and T478K of the S-protein and T402I of NSP4, which increased with VOCs Delta and were recruited by VOC Omicron. Free-standing marker P681H shared by the VOCs Alpha and Omicron was lost in October-November 2021 but was then regained ( Figure 2C).

### Latitude-linked patterns of seasonality

**Figure 6.**
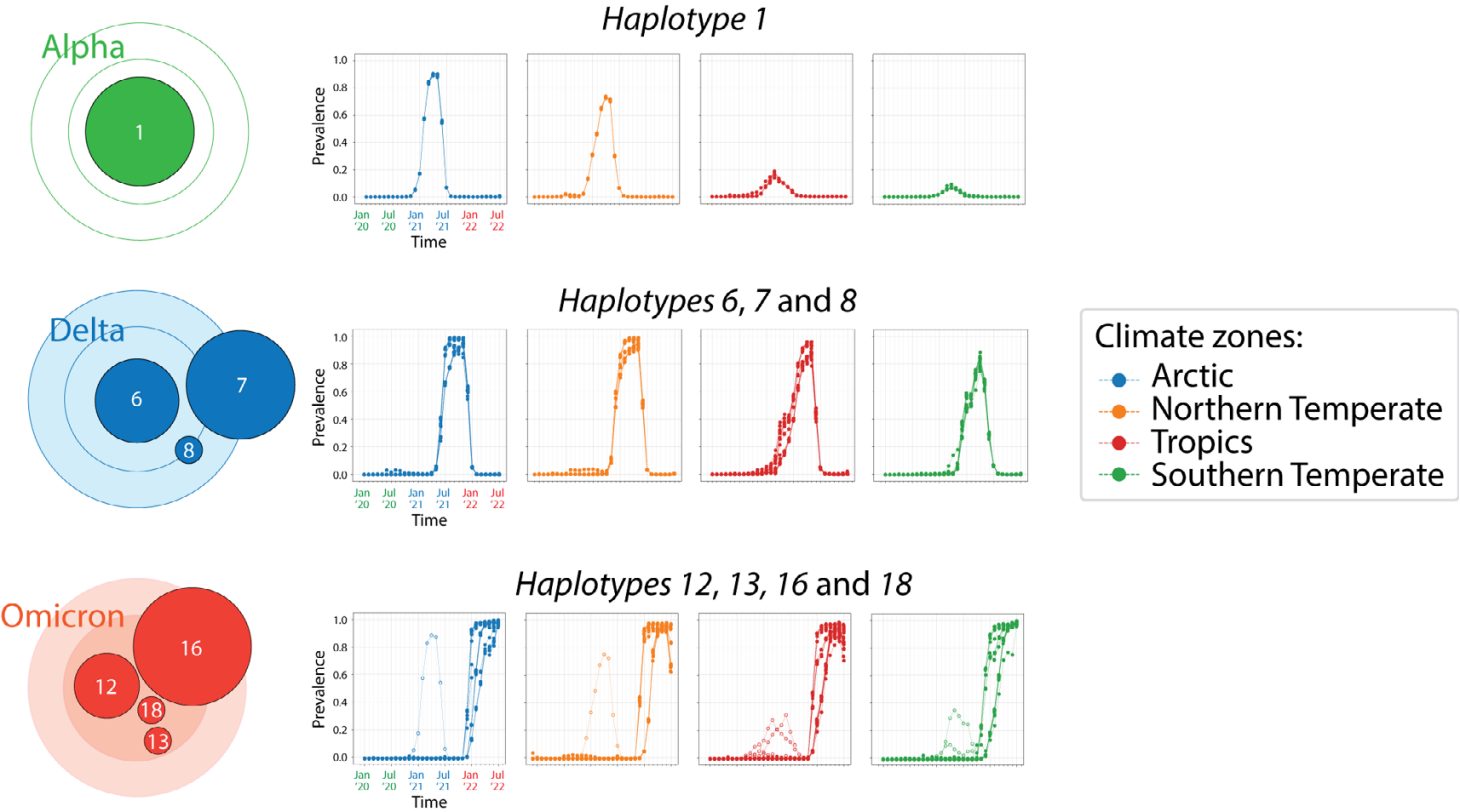
Patterns of mutation accumulation in core haplotypes of VOCs revealing seasonal behavior. Separate plots describe overlaps of mutation accumulation curves for the four climate zones. Open symbols describe regions of the timeline unrelated to the VOC of reference.

A global analysis of COVID-19 seasonal behavior during the early stages of the pandemic showed that effective disease transmission was restricted to a 30° to 60° latitude corridor in both the Northern and Southern Hemispheres (reviewed in Caetano-Anollés *et al.,* 2022). An initial study however failed to reveal an association between genomic and epidemiology data ( Burra *et al.*, 2021), perhaps because the focus was global genomic change levels during the first wave of the pandemic. In contrast, overlaps of accumulation plots for mutants belonging to individual haplotypes already uncovered distinct latitude-dependent accumulation trends in our study ( Figure 2B). These trends dissected the tropics from the temperate regions of the world. To better visualize these accumulation patterns we selected haplotypes that coalesced into the highly cohesive cores of the three haplotype subnetworks in Figure 5. We excluded haplotypes arising before the first appearance of VOCs ( *haplotypes 2* and *5*) and those highly variable shared by the VOCs Alpha and Omicron, focusing instead on haplotypes with minimum decoupling. The haplotype core of VOC Alpha involved only *haplotype 1*, while those of VOC Delta and Omicron involved three and four haplotypes, respectively. Overlap plots showed separate patterns of emergence and decoupling for corridor (Northern and Southern Temperate) versus non-corridor (Tropics and sometimes Arctic) climatic regions ( Figure 6). The 12 markers of the VOC Alpha core behaved monolithically in Arctic, Northern Temperate and Southern Temperate regions, showing a single pattern of accumulation that peaked in May 2021. In contrast, the rise of the core in the Tropics revealed a multiplicity of accumulation patterns, with at least four distinct curve types. As described above, prevalence levels of *haplotype 1* markers in the Northern Hemisphere were considerably higher than those in the Southern Hemisphere. The 22 markers of the VOC Delta core also accumulated into a single peak, although the peak was broader (spanning August-November 2021) and often reached close to 100% prevalence levels in the Northern Hemisphere. Again, the rise of the core in the Tropics showed a multiplicity of accumulation patterns, with at least seven distinct curves leading to a single peak. This noisy emergence contrasts with a rather cohesive emergence of the constellation in the other climatic regions. Finally, the 32 markers of the VOC Omicron core again showed a clear distinction between the corridor and non-corridor regions. The Northern and Southern Temperate zones showed two clear steep accumulation curves that were quite variable and offset by a 2-month period. As expected, the rise of VOC Alpha occurred earlier in the Southern Temperate zone given its presumed South African origin. In contrast, the Tropic and Arctic zones showed three to four main accumulation routes, each of which had much closer origins and were less steep. A comparison of the number of non-overlapping monthly prevalence counts (symbols in Figure 5) during the November 2021 to July 2022 period revealed that the Northern and Southern Temperate regions had 48 and 57 counts, respectively, while those of the Tropics and Arctic regions had 77 and 61 counts, respectively. The difference shows higher decoupling (i.e. decreased coalescence) occurring in non-corridor regions of the world.

### Defining a protein network of haplotype interactions

**Figure 7.**
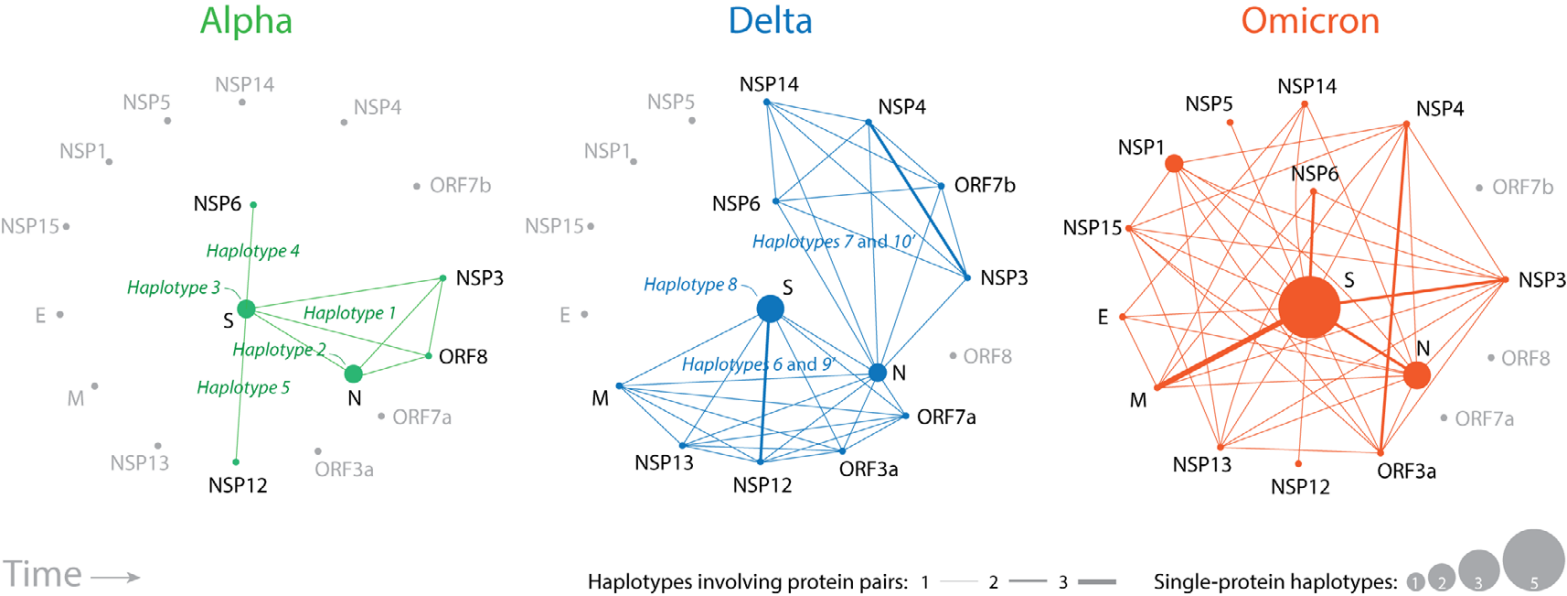
Evolving network diagrams describing SARS-CoV-2 protein interactions mediated by haplotypes. Nodes are proteins and lines in the graph are protein interactions manifesting as joint protein presence in a haplotype. Node size is proportional to the number of haplotypes harboring markers that affect only one protein. Line width is proportional to the number of haplotypes sharing the same pair of proteins. Larger nodes and thicker lines highlight the significance of protein roles.

Mutations gradually alter the proteins of the virus by introducing errors during RNA replication or repair, presumably stochastically in the viral population. Once haplotypes appear, mutations are coordinated to maintain stable protein structures through compensation, modulate protein translation and localization, or benefit their overall functionality. While haplotypes of the VOCs Alpha and Delta constellations affected (proportionally) a relatively diverse set of proteins, those of VOC Omicron were enriched in mutations impacting the S-protein. To explore this shift in more detail we constructed ‘protein interaction networks’ illustrating how interactions within or between proteins in haplotypes altered protein structure and function ( Figure 7). In these networks, nodes depicted proteins and links depicted interactions between them, that is, joint protein presence in a haplotype of a VOC constellation through active coordination. The node size is made proportional to the number of haplotypes harboring mutant markers affecting only one protein (e.g. *haplotypes 2* and *3*). The width of links is proportional to the number of haplotypes sharing the same pair of proteins. Thus, larger nodes and thicker lines of the network highlighted a more important role of proteins and their associated protein interactions at inter- and intramolecular levels.

Haplotype interactions of VOC Alpha ( Figure 7, green lines) involved the structural S-protein and N-protein molecules, few nonstructural proteins (NSP3, NSP6, NSP12), and the ORF8 accessory protein. VOC Delta expanded the interaction repertoire by constructing two subnetworks connected by the N-protein, one involving the S-protein, M-protein, NSP12, and NSP13 and the accessory proteins ORF3a and ORF7a (mediated by *haplotypes 6* and *9’*) and the other one involving NSP3, NSP4, NSP6, NSP14 and ORF7b (mediated by *haplotypes 7* and *10’*). VOC Omicron integrated these two subnetworks with additional structural and nonstructural proteins. Note the increasing importance of the M-protein, NSP3 and NSP6 proteins, and the intramolecular interactions of the S-protein and N-protein. It is also noteworthy how interactions between structural proteins were enhanced following each VOC replacement.

### Haplotype markers were either spread or clustered along the S-protein sequence

**Figure 8.**
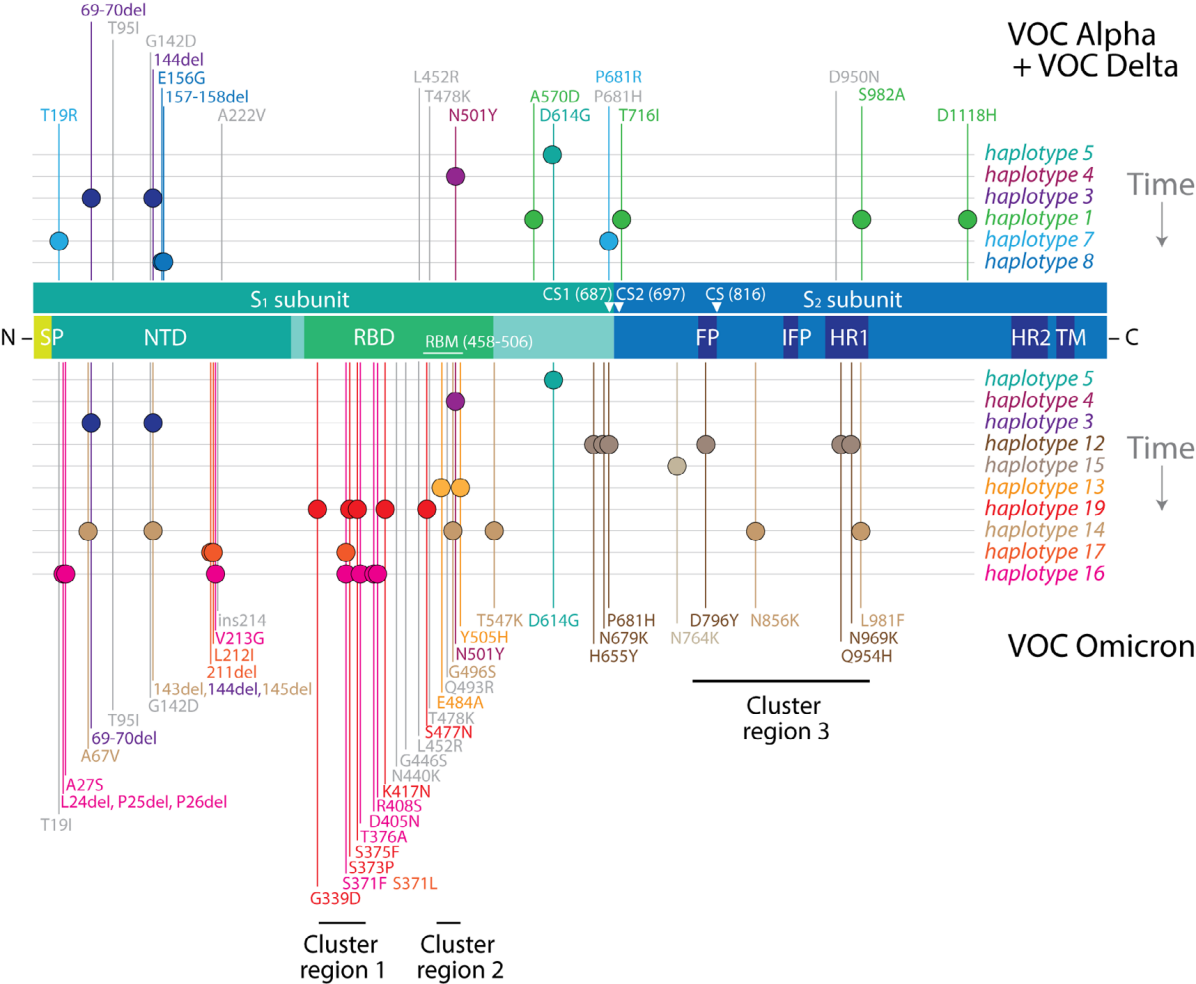
Haplotype markers clustered along the S-protein sequence. The diagram maps mutations onto the amino acid sequence of the S-protein molecule, from the N- to the C-terminus, with markers specific to VOCs Alpha and Delta indicated at the top and those specific to VOC Omicron at the bottom. Mutations in VOC Omicron cluster in groups according to haplotype and are enriched in immune evasion functions associated with the RBD region. Mutations in haplotypes 1, 12, and 14 spread through the molecule and likely make up networks of allosteric interactions. Clusters 1, 2, and 3 represent mutation targets at codon sites known to be either negatively selected or evolving under no detectable selection in non-Omicron sequences ( Martin *et al.*, 2022). Markers highlighted in grey represent free-standing mutations. SP, signal peptide; NTD, N-terminal domain; RBD, receptor-binding domain; RBM, receptor-binding motif; CS, cleavage site; FP, fusion peptide; IFP, internal fusion peptide; HR1, heptad repeat 1; HR2, heptad repeat 2; TM, transmembrane domain.

We explored the location of mutations in the S-protein for two reasons: the centrality of the S-protein in viral infection and the massive enrichment of mutations in VOC Omicron. We asked if haplotype markers were spread or clustered along the S-protein sequence. Remarkably, we found two distinct behaviors ( Figure 8). Markers of *haplotypes 1*, *12*, and *14* were spread through large swaths of the sequence, especially those of *haplotype 14.* These haplotypes share the unique property of impacting fusogenic regions of the S _2_ subunit. In contrast, markers of the rest of the haplotypes were significantly clustered. For example, markers of *haplotypes 4, 13*, *16,* and *19* localized to the RBD, while *haplotypes 4* and *13* targeting exclusively the receptor-binding motif (RBM) of the domain. Similarly, markers of h *aplotypes 3*, *7* and *8* localized to the NTD. The most recent haplotypes of VOC Omicron, *haplotypes 16* and *17*, were particularly interesting because they targeted both the NTD and RBD domains. We hypothesize that haplotypes with markers that are spread involve allosteric interactions that regulate the functional activities of the S-protein, including receptor-binding, membrane fusion, and interactions with other proteins. Haplotypes with clustered mutations appear to have more direct roles, impacting the binding activities of the NTD and RBD regions of the molecule. Free-standing mutations ( Figure 8, labeled in grey) were particularly spread throughout the S _1_ subunit and often tightly associated with haplotypes linked to these regions, suggesting a tendency towards coalescence. For example, the RBM-linked S478K and Q493R markers were closely clustered with markers of *haplotypes 4* and *13* while at the same time differing in patterns of accumulation ( Figure 8). Mapping haplotype history also uncovered interesting trends. Except for *haplotype 4*, all VOC Alpha and Delta haplotypes failed to target the RBD region. Instead, the focus was flexible regions between the two subunits of the S-protein or the seasonality-linked NTD region. The arrival of VOC Omicron shifted the mutational spectrum from those regions first to RBD and then to the C-terminal region of NTD.

### *Ab initio* modeling of the structure of the M-protein uncovers haplotype-linked structural change

**Figure 9.**
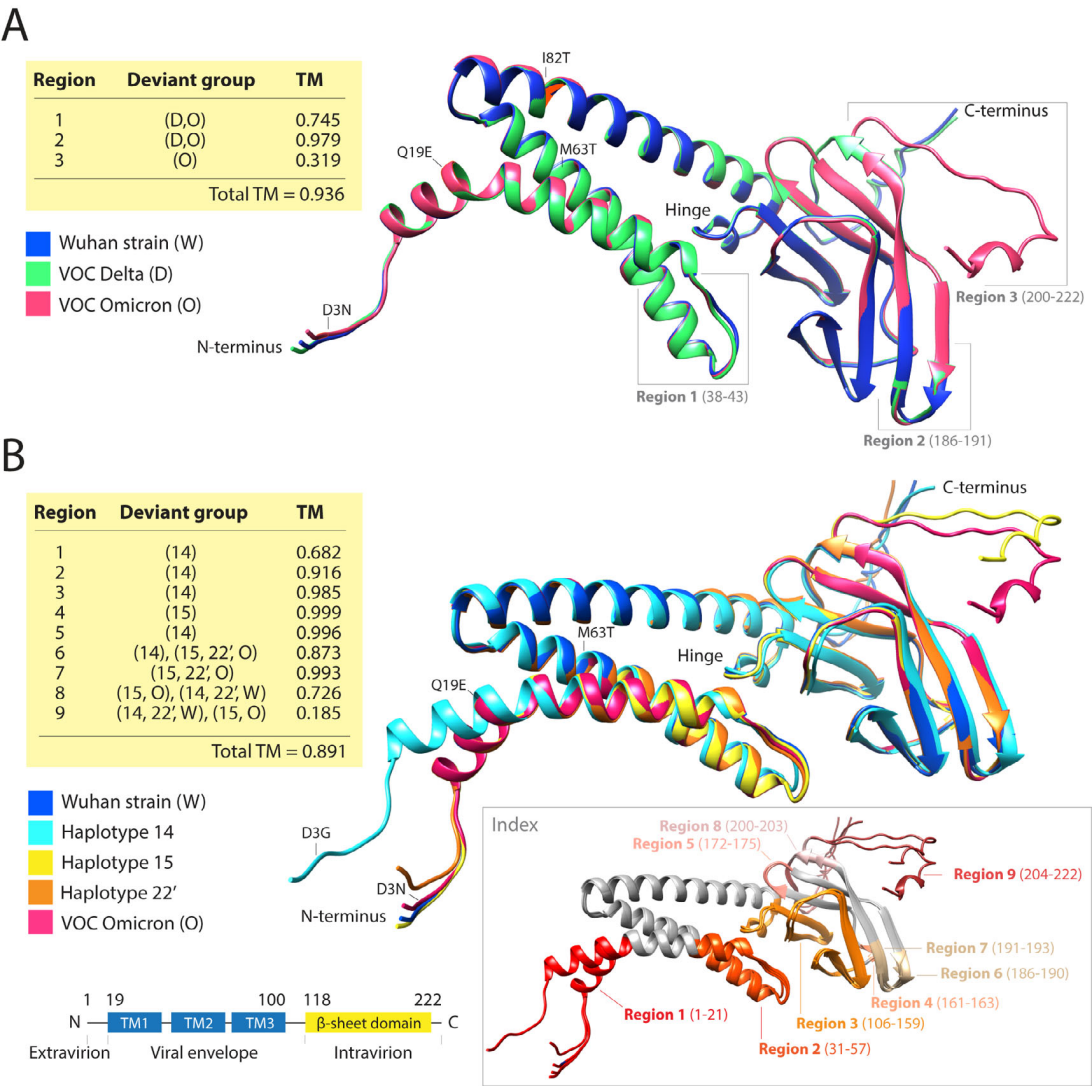
AlphaFold2 *ab initio* modeling of evolving atomic structures of the M-protein. The structures of reference and mutated variants of the M-protein were modeled directly from their sequences using AlphaFold2. Their structures were then aligned, and regions exhibiting structural differences (indexed in the structural models) were further examined qualitatively by determining deviant groups and quantitatively using template modeling (TM) scores. (A) Structural alignment of M-protein molecules of the reference Wuhan strain and those typical of VOC Delta and VOC Omicron. The locations of mutations and regions with structural differences are indicated. The table describes deviant groups and TM-scores for the different regions. (B) Structural alignment of modeled molecules evaluating structural effects of mutations of the VOC Omicron constellation and related haplotypes. The left inset at the bottom shows a schematic view of M-protein domain organization mapped onto the sequence. The three transmembrane helices (TM1, TM2 and TM3) make up a bundle and multiple strands make up the C-terminal β-sheet domain. The right inset colors structural deviant regions directly onto the aligned structures.

We used the local ColabFold implementation of the AlphaFold2 *ab initio* artificial intelligence pipeline to model the atomic structure of the M-protein. Our goal was to illustrate how modeling with deep learning tools directly from amino acid sequences can help dissect the impact of mutations on the structure of viral proteins. We focused here on the M-protein because of its increasing relevance ( Figure 6); an exhaustive *ab initio* modeling exploration of the entire SARS-CoV-2 proteome will be reported elsewhere. Since only VOCs Delta and Omicron exhibit haplotypes altering the M-protein ( Figure 6), we first compared the modeled structures of the Wuhan reference strain against those of VOC Delta and Omicron strains ( Figure 9A) and then dissected the individual effects of haplotypes on molecular structure ( Figure 9B). Qualitative assessments were complemented with quantitative measurements of structural differences, including the calculation of TM-scores ( Zhang & Skolnick, 2004) for residues in structural deviant regions. VOC Delta differs from the reference strain by one mutation, I82T of *haplotype 7.* Conversely, VOC Omicron differs from the reference strain by four mutations: D3G of *haplotype 14*, Q19E and A63T of *haplotype 15*, and D3N of *haplotype 22’.* D3G and D3N are located in an intrinsically disordered region of the molecule ( Zhang *et al.*, 2022b). All mutations were located in the N-terminal region of the molecule, but most structural effects were felt downstream in the C-terminal β-sheet domain region.

Comparing the VOC Delta and Omicron molecules to those of the reference strain revealed notable structural changes ( Figure 9A). Significant and equal structural divergences occurred in the linker spanning the first and second transmembrane helices of the N-terminal triple-helix that delimits the ‘viral envelope domain’ bundle ( *region 1*; residues 38-43) in both VOC molecules. Similarly, more limited structural divergences affected the β-strands anchoring the terminal loop of the C-terminal ‘intravirion domain’ ( *region 2*; residues 186-191). In sharp contrast, the most distal C-terminal region of the ‘intravirion domain’ ( *region 3*; residues 200-222) was the most affected, especially in the VOC Omicron variant, with shortening of the last helix by one residue and reformation of the coil into a helix at the C-terminus (residues 218-220). The TM-scores for *regions 1* and *3* were significantly lower than the average of the whole structure (0.936), indicating that those regions were significantly impacted by structural change. TM-scores range from 0 to 1, with 1 indicating perfect structural match and values below 0.2 indicating structural matches should be considered random ( Zhang & Skolnick, 2004).

The individual effects of VOC Omicron haplotypes on the M-protein structure were even more revealing ( Figure 9B). We identified nine regions with significant structural differences. Only two of these regions affected the N-terminal ‘intravirion domain’ and only four ( *regions 1*, *6*, *8*, and *9*) had TM-scores lower than the average TM-score of the entire structure (0.891). Remarkably, we observed that the VOC Omicron mutant constellation balanced the effect of the individual haplotypes. In *region 1*, VOC Omicron counteracted the effect of *haplotype 14*, which caused a conformational shift that twists the first helix of the ‘viral envelope domain’ at amino acid residue 21 and reduced the TM-value to 0.682. In *region 6*, VOC Omicron and *haplotypes 15* and *22’* balanced *haplotype 14* and its ability to lengthen a β-strand anchoring the terminal loop of the ‘intravirion domain’ (matching deviant *region 2* in Figure 9A). This effect reduced the TM-score to 0.873. In sharp contrast, *haplotypes 14* and *22’* were unable to counteract the shortening of the last helix by 1-2 residues and the reformation of the coil into a helix in *regions 8* and *9* (matching deviant *region 3* in Figure 9A) caused by VOC Omicron and *haplotype 15.* These effects were more impactful, reducing TM-scores to 0.726 and 0.185, respectively. Remarkably, the highly-conserved hinge region (residues 106-116 of *region 3* in Figure 8B) was hardly affected by the mutations (especially *haplotype 14*), leading to a drop in the TM-score to only 0.985. This hinge controls conformational changes of M-molecules affecting virus formation during infection ( Arndt *et al.*, 2010). Overall, the mutant make-up of VOC Omicron and *haplotype 15* appear major contributors to the distortion of the C-terminal ‘intravirion domain’ while maintaining the structure of the ‘viral envelope domain’ and the hinge region that connects the two domains of the molecule. Thus, mutant VOC constellations balance the more extreme effects of individual haplotypes on protein structure. A similar landscape of structural frustration, which permeates the rise of haplotypes and their make-up, exists in other viral proteins, which will be reported elsewhere.

**Figure 10.**
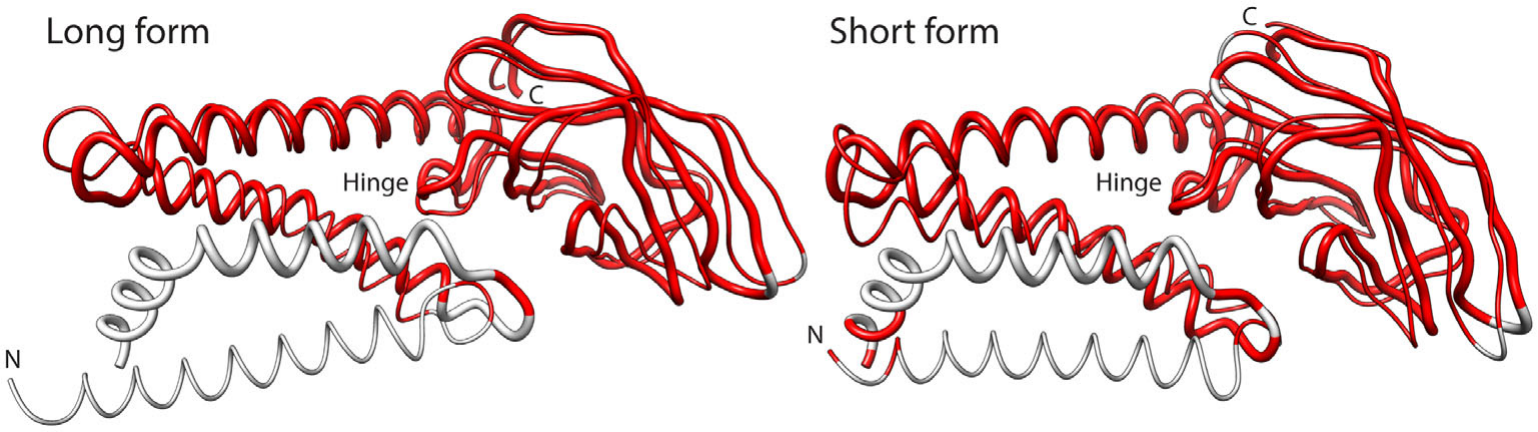
Alignment of long and short forms of the M-protein acquired by cryo-EM (thin backbones) to AlphaFold2 predicted structures (thick backbones). The superimposed regions with residues separated by distances less than 5 Å colored in red.

We confirmed the validity of our AlphaFold2 predictions with experimental M-protein models recently obtained by cryo-EM ( Zhang *et al.*, 2022b). The study used two monoclonal antibodies as fiducial markers (M/Fab-E and M/Fab-B) to capture two different conformations of the M-protein in the complexes, a long form and a short form. Aligning our models to the long and short structures resulted in TM-scores of 0.693 and 0.729, GDT_TS scores of 0.606 and 0.622, and superposition RMSDs of 2.82 Å and 2.59 Å for 161 and 165 aligned C _α_ residues (< 5 Å distances), respectively ( Figure 10). These scores support models with accurate topologies and very good structures for membrane proteins. Note that most structural deviations occurred in the first 30 aligned residues of the M-protein, that is, in the most N-terminal transmembrane helix that holds mutations in all VOC Omicron haplotypes. Also, note that the short form showed better alignment scores. This is particularly relevant for the functional role of the modeled structures. Tomographic and other approaches have associated the long form of the M-protein with rigidity, narrow curvature of the viral envelope as formed in the endoplasmic reticulum, and increased recruitment of S-protein and N-protein during virion assembly ( Neuman *et al.*, 2011). In contrast, the short form was associated with flexibility and decreased recruitment capabilities. This suggests that our structural predictions better match M-forms that decrease the budding and efficiency of virion assembly.

## Discussion

### VOC emergence

There appears to be no ‘transparent’ path of transmission from viral ancestors to VOCs nor an explanation for the massive mutant constellation that characterizes the current VOC Omicron wave of the pandemic ( Kupferschmidt, 2021; Mallapaty, 2022). Indeed, the appearance of VOC Omicron in Botswana and South Africa and its fulminant and massive spread throughout the world has been both puzzling and unanticipated ( Viana *et al.*, 2022). Here we offer an explanation grounded in an analysis of about 12 million genome sequences of the virus.

First, we show that many VOC mutations were already present in various combinations during the first wave of the pandemic and were recruited piecemeal to form increasingly large constellations. For example, more than half of S-protein markers of VOC Omicron were already present on November 2020, seeding the rise of 93% of S-protein-containing haplotypes ( Table 2). These results align with a previous analysis that revealed a significant number of S-protein markers were present in VOC constellations, sometimes as novel 10-mutant combinations ( Caetano-Anollés *et al.*, 2022). Thus, haplotype primordia were already emerging very early in the evolving viral population.

Second, we show that the rise of VOC markers did not occur monolithically or in a coordinated manner worldwide. Instead, idiosyncratic patterns of marker accumulation were evident in latitude-dependent regions of the world. These distinct accumulation behaviors allowed construction of a chronology of haplotypes, all of which ultimately coalesced into VOC constellations ( Figure 2). Thus, the rise of constellations resembled a process of viral emergence in which haplotypes and free-standing markers were integrated piecemeal (most likely by recombination) to facilitate infection capabilities, viral transmission, and epidemic spread. Note that virus emergence is an eco-evolutionary process that rapidly samples the genomic sequence space of the viral quasispecies while locating high-fitness combinations of mutations ( Dennehy, 2017). Our previous mutation accumulation study of the viral populations of Australia also supported the emergence scenario, especially because of the very early, uncoordinated, and massive displacement of mutations of VOC Delta by those of VOC Omicron occurring in a country that had not yet reopened international and regional borders and had one of the toughest restriction policies on the planet (lockdowns, extensive contact tracing, mandatory vaccination, and strong quarantine restrictions) ( Tomaszewski *et al.*, 2022). The emergence scenario also explained the puzzling fact that VOC Omicron was first detected in samples collected November 11-16, 2021 in the African continent. However, its prevalence in Australia increased massively some weeks after being first reported in the continent on November 28-29, 2021. Physically, there could be no trans-continental path of transmission that would be so massive and efficient.

Third, we dissected the VOC Omicron constellation into 16 haplotypes harboring 78 mutant markers affecting almost half of the SARS-CoV-2 proteome (14 proteins). Haplotypes were identified through coordinated patterns of marker accumulation in plots describing monthly increases and decreases of mutation prevalence along four latitude corridors of the world ( Figure 2). A worldwide haplotype network illustrated how haplotypes coalesced into VOC constellations ( Figure 5). Our haplotype analysis matched that of Australia ( Tomaszewski *et al.,* 2022), revealing similar haplotype recruitments, dynamic behaviors, and putative molecular interactions operating at the proteome level. It also showed increased constellation coalescence, an expected outcome given that our worldwide latitude dissection was less coarse-grained than that of a single continent in the Southern Hemisphere. Remarkably, many haplotypes were decoupled by seasonality when marker accumulation in latitude corridors failed to overlap in prevalence plots ( Figure 6). This highlights the highly dynamic nature of the evolving viral population worldwide and the existence of an important seasonal effect that is genetically imprinted in the viral quasispecies.

Our results are not only in line with findings for Australia ( Tomaszewski *et al.,* 2022) but also those for the United States ( Tasakis *et al.*, 2021; 62,211 genomes; January 2020–April 2021) and England ( Vöhringer *et al.*, 2021; 281,178 genomes; September 2020–June 2021). Results are also compatible with a worldwide longitudinal study of mutations targeting the S-protein and the NSP12 polymerase ( Showers *et al*., 2022; 437,006 genomes; January 2020–January 2021). The studies suggested that the viral genome was evolving dynamically to become more structured. In all cases, mutations accumulated in bursts, often followed by sweeps, while haplotypes and VOC constellations were generated in waves. Major global shifts in the selective landscape and possible convergence between lineages likely drove the rise of constellations ( Martin *et al.*, 2022), with viruses changing in response to hosts and mitigations (e.g. vaccination). Mutational changes were particularly exacerbated when genetic drift and ‘super spreader’ events in a sea of mutational bursts drove infrequent mutations to prominence ( Choi *et al.*, 2020). Other explanations of VOC origin ( Kupferschmidt, 2021; Mallapaty, 2022) appear less compatible with the global latitude-dependent accumulation patterns we here uncovered, including founder effects or bottlenecks occurring in single chronically-infected ( Ghafari *et al.*, 2022; Magiorkinis, 2023) or HIV-infected ( Tarcsai *et al.*, 2022) patients, origins in non-human animal hosts (e.g. mouse; Wei *et al*., 2021), or hidden spread in multiple hosts that would massively distribute the newly emergent variants ( Ghafari *et al.*, 2022; Magiorkinis, 2023). All of these proposals stress local rather than global mechanistic explanations. While a combination of local and global mechanisms is likely, patterns of VOC emergence may result from constraints operating worldwide at different time scales on the evolving viral quasispecies, including those imparted on proteins by mappings between the spaces of possible sequences and structures ( Dennehy, 2017), of which we have limited understanding ( Ferrada & Wagner, 2010). We note that the ‘cryptic spread’ of SARS-CoV-2 lineages has been detected in wastewater, suggesting an evolutionary convergence ( Gregory *et al.*, 2022). This spread appears to be occurring undetected by genomic surveillance within a highly diverse genetic background.

### Seasonality

Seasonal variation impacts the persistence of living beings ( Dayton, 2019). It influences the abundance and distribution of organisms and viruses through space and time. The seasonal behavior of SARS-CoV-2 has become a topic of great interest ( Kissler *et al.*, 2020), especially because seasonality could assist in formulating actionable pandemic responses. Coronaviruses are considered ‘winter viruses’ and are expected to exhibit seasonal behavior ( Nickbakhsh *et al.,* 2020). In fact, significant statistical associations exist between seasonal variations and the survival and transmissibility of the virus; higher latitude, colder temperatures, and lower humidity have all been linked to higher COVID-19 incidence in local and global epidemiological analyses (e.g., Demongeot *et al*., 2020; Sajadi *et al.*, 2020; Burra *et al*., 2021; Liu *et al.*, 2021). Effective disease transmission appeared restricted to a 30° to 60° latitude corridor in both the Northern and Southern Hemispheres with data suggesting disease outbreaks follow those of influenza, moving across the planet along a sinuous curve parallel to the solar solstice ( Hope-Simpson, 1981; Deyle *et al.*, 2016). A molecular link between environment and physiology that would explain seasonal cycles has remained elusive despite over 150 years of research on epidemic calendars ( Grenfell & Bjørnstad, 2005; Martinez, 2018). A recent worldwide mutation prevalence study, however, revealed that mutation bursts affecting the galectin-like structure of the NTD region of the SARS-CoV-2 S-protein followed hemisphere-related patterns ( Caetano-Anollés *et al*., 2022). Furthermore, the accumulation of haplotypes involving the S-protein and other proteins was also latitude-dependent ( Tomaszewski *et al*., 2022). We here extend these initial analyses by uncovering significant latitude-dependent differences in VOC haplotype accumulation ( Figure 2) and decoupling of core haplotypes in non-corridor latitude regions ( Figure 6). These findings further support seasonal cycle-mediated molecular interactions confirming viral genetics mediates seasonal behavior.

The role of the galectin-like fold of the NTD region of the S-protein is particularly significant. Both the NTD and RBD regions recognize and bind to sugars and other host cell receptors, enabling viral attachment and infection ( Pourrajab, 2021). They also present N- and O-linked glycosylation sites that act as a ‘glycan shield’ to camouflage the virus from host defenses. Galectins are evolutionarily conserved glycan-binding effector proteins that regulate various processes, including cellular and extracellular interactions, pathogen recognition, and inflammation ( Dings *et al.*, 2018). These roles involve binding to the carbohydrate moieties of glycoconjugates present on cell surfaces, reducing endocytosis by forming lattices, and regulating immunity signals ( Pourrajab, 2021). Galectins can either facilitate or avoid infections of a wide range of pathogens ( Ayona *et al*., 2020). The camouflaged galectin-like structures of the S-protein likely help viral attachment and immune evasion, possibly by impersonating host galectins ( Wang *et al*., 2020). For example, the NTD domain (but not other S-protein regions) was shown to activate human monocytes to produce cytokine cascades responsible for the acute respiratory distress syndrome (ARDS) of COVID-19 in vitro ( Schroeder & Bieneman, 2022). This activation mimics the effect of host galectin-3, which facilitates activation of immune cells. It is noteworthy that administration of SARS-CoV-2 galectin-like inhibitors reportedly decreased viral loads in patients ( Sethi *et al*., 2020). These studies stress the central role that galectins play in modulating COVID-19 infection.

Galectin moieties act as environmental sensors when their activities are modulated by temperature. Recent evidence of such a role comes from an unexpected source, the coral reefs of the Pacific and Indian Oceans and the devastation caused by coral bleaching. Rising ocean water temperatures and other factors of global change disrupt the healthy symbiosis between scleractinian corals and dinoflagellates and the clearance of pathogens (e.g., Kleypas *et al.*, 2015). A number of lectins, including galectin proteins with antimicrobial immunity functions, have been implicated in thermal and disease stress responses of coral communities (e.g. Ricci *et al.*, 2019). Remarkably, galectins of scleractinian ‘cauliflower’ corals acted as environmental sensors, recognizing and clearing coral pathogens when temperatures were optimal (25 ^o^C and 30 ^o^C) while allowing pathogen survival at lower temperatures ( Wu *et al.*, 2019). A similar temperature dependence was observed when studying the flexibility of the SARS-CoV-2 S-protein with all-atom molecular dynamics simulations ( Rath & Kumar, 2020). The NTD was much more mobile than the RBD, which exhibited only a flexible RBM. Increasing temperatures made the NTD top layer residues less solvent exposed while closing the flexible RBM of the RBD in the trimer. All of these residues involved mutations highlighted in Figure 8. These conformational changes sealed the visibility of the trimeric pore, burying the receptor binding residues necessary for ACE2-binding, and inactivating (perhaps reversibly) the S-protein with temperatures above 40 °C. These results suggest that NTD binding properties depend on flexibility optimized by environmental change.

### Three phases

Caetano-Anollés *et al.* (2022) studied the appearance of mutation bursts affecting the S-protein and concluded that the COVID-19 pandemic followed three successive phases, the first driven by an interplay of protein flexibility and rigidity, the second by environmental sensing, and the last phase by immune escape ( Figure 1A). The timeline of haplotypes ( Figure 2) and their clustering along the S-protein sequence ( Figure 9) support such an interpretation. The first haplotypes impacted the flexibility of the S-protein. The D614G mutation of *haplotype 5* disturbed hydrogen bonding interactions between the S _1_ and S _2_ subunits of different protomers of the S-protein as well as contacts with the FP region that are necessary for membrane fusion ( Korber *et al.*, 2020; Yurkovetskiy *et al.*, 2020; Xu *et al.*, 2021). Following the rise of *haplotype 2*, which affected the intrinsically disordered linker region of the N-protein ( Tomaszewski *et al.*, 2020), *haplotype 1* introduced four S-protein markers (A570D, T716I, S982A and D1118H) in regions of increased protein disorder that mostly affected the C-terminal S _2_ subunit ( Caetano-Anollés *et al.*, 2022). *Haplotype 3* introduced deletions (H69del, V70del, and Y144del) located in the NTD region holding the galectin-like structure associated with environmental sensing and seasonal behavior ( Caetano-Anollés *et al.*, 2022). Finally, *haplotype 4* and a free-standing mutation introduced two crucial markers (N501Y and P681H) impacting the immunogenic RBD region responsible for ligand binding (e.g., ACE2). Note that N501Y involved one of six contact residues of RBD known to increase both ACE2 receptor affinity and virulence ( Starr *et al*., 2020), while P681H altered one of four residues comprising the insertion that creates the S1/S2 furin cleavage site between the S _1_ and S _2_ subunits ( *see* Harvey *et al.*, 2021). VOC Delta haplotypes appearing at the beginning of the immune escape phase focused for the most part on mutations in other proteins but continued to involve the environmental sensing NTD region (T19R of *haplotype 7* and deletions E156G, F157del, and R158del of *haplotype 8*) and the RBD site (P681R marker of *haplotype 7*). Similarly, free-standing markers also impacted the NTD (T96I, G142D, A222V) and RBD (L452R, T478K) regions. VOC Omicron recruited most of these markers via *haplotypes 3*, *4*, and *12* but then massively acquired mutations in the RBD, NTD and S _2_ subunit regions via several additional haplotypes, with the novelty that two of these haplotypes affected both the sensing and immunogenic regions ( *haplotypes 16* and *17*) and one affected all three regions ( *haplotype 14*) ( Figure 8). Thus, tight networks of intramolecular interactions appeared to unify change in all functional regions of the S-protein as the pandemic advanced. Note that the entire constellation of VOC Omicron is under gene-wide positive selection ( Viana *et al*., 2022) and that many mutations arose from molecular interactions that were collectively adaptive ( Martin *et al*., 2022). A group of 13 was clustered into three regions of the S-protein (horizontal lines in Figure 8) mapping to *haplotypes, 12*, *13*, *16,* and *19.*

### Protein interactions

Networks of protein interactions describe haplotype-mediated protein communications that impact the structure and function of proteins ( Figure 7). Typically, these molecular interactions involve direct communications within (e.g., allosteric control) or between molecules (e.g., protein-protein interactions), or indirect communications through shared or linked functions. We traced how protein networks changed with every VOC replacement to study evolutionary constraints and pathways of evolutionary optimization. As mutant constellations evolved, protein interaction networks became organized around the S-protein, N-protein, and M-protein molecules via intramolecular and intermolecular relationships ( Figure 7). This is an expected outcome. Intraviral interactions between these three structural proteins are essential for hijacking the host’s cellular machinery ( Siu *et al*., 2008; Fehr & Perlman, 2015). The multifunctional spike glycoprotein plays roles in target recognition (e.g. viral attachment to cell receptors, cellular tropism), cellular entry (viral fusion), and endosomal escape (e.g., capsid assembly), not to mention roles in transmissibility and virulence ( Huang *et al*., 2020; Magazine *et al*., 2022). Its highly immunogenic properties have made the S-protein a target for drug and vaccine development ( Harvey *et al.*, 2021). The N-protein packages the RNA genomes but plays critical roles in replication, virion assembly, and regulation of the viral life cycle ( Bai *et al*., 2021). Proteolysis of the intrinsically disordered linker that separates the two domains of the N-protein generates at least five proteoforms that bind structured RNA and provide regulatory and immunogenic functions ( Lutomski *et al.*, 2021). The transmembrane M-protein is crucial for virus assembly and membrane budding ( Fehr & Perlman, 2015). It consists of an N-terminal ‘viral envelope’ ectodomain made of three transmembrane helices and a C-terminal globular ‘intravirion’ endodomain. The endodomain interacts with the N-protein, S-protein and RNA molecules for oligomerization, RNA encapsulation, and mature virus particle formation but also with the E-protein with the help of the two most central transmembrane helices ( Hsieh *et al.*, 2008; Zhang *et al.*, 2022b). The M-protein localizes in the endoplasmic reticulum–Golgi intermediate compartment and recruits other viral structural proteins ( Fehr & Perlman, 2015). Molecular dynamic and docking simulations of SARS-CoV-2 structural proteins recently revealed that the M-protein acts as a receptor, while the S-protein, N-protein, and E-protein act as protein ligands ( Kumar *et al.*, 2021). This is in line with cryo-EM, tomography and statistical evidence supporting the central role played by the M-protein in virion assembly ( Neuman *et al.*, 2011). Remarkably, all of these interactions materialize in the evolving haplotype-mediated interaction networks.

Similarly, domains and linkers of the N-protein interact with a number of proteins, including the M-protein ( Lu *et al.*, 2021) and the multidomain NSP3 papain-like protease ( Hurst *et al.*, 2013). NSP3 processes viral polyproteins, forms the viral replicase-transcriptase complex with other NSPs and RNA, and antagonizes the host innate immune response ( Lei *et al.*, 2018). The N-protein and NSP3 connection was established early via multiple markers of *haplotype 1* in VOC Alpha, *haplotype 6* in VOC Delta, and *haplotype 16* in VOC Omicron ( Figure 7). The functionalities of the S-protein, nucleocapsid and NSP3 proteins coalesce in the VOC Omicron network, highlighting their well-known centrality in viral transmissibility, disease severity, and immune escape. Their role is further enhanced by interactions with the autophagy-associated NSP6 protein. NSP6 induces formation of multimeric sensor proteins (inflammasomes) and autophagosomes, mediating caspase-1 activation and secretion of pro-inflammatory cytokines known to induce inflammatory cell death ( Cottam *et al.*, 2011; Sun *et al.*, 2022). Aberrant activation of inflammasomes can cause cascades leading to the severe respiratory syndromes of SARS-CoV-2 ( Rodrigues *et al.,* 2021). NSP6 network connections were established early via *haplotype 4* in VOC Alpha and then VOC Omicron, *haplotype 6* in VOC Delta, and *haplotype 14* in VOC Omicron ( Figure 7). This prompts evaluation of how the new constellations are softening aberrant immunity activations.

### Molecular structure

The effect of mutations on the protein sequence must be linked to effects at atomic structure level to dissect the functional significance of individual haplotypes and constellations. Three main strategies model protein structure: homology modeling, fold recognition, and *ab initio* methodologies. Homology modelling and fold recognition rely on the existing sequence and folded structure data and are rather comparative in nature. These methods can be limited in their ability to accurately predict the true 3-dimensional structure of novel proteins, especially in molecular systems subjected to fast mutation rates. *Ab initio* methods however do not use pre-existing knowledge. Instead, they build models directly from amino acid sequences and the stoichiometric constraints of those sequences. Such an approach is especially useful for modeling proteins with low homology. We modeled the 3-dimensional atomic structures of mutant SARS-CoV-2 proteins defining haplotypes and constellations with AlphaFold2 ( Jumper *et al.*, 2021). AlphaFold2 is the star of the last two biannual structure prediction experiments (CASP 14 and 15). Its deep learning algorithm makes fast atomic structural predictions with levels of accuracy that are within the margin of error of experimental structure determination methods. Crucially, this reduces reliance on traditional crystallographic methods that are time-consuming. Because of the central role that the M-protein plays in delimiting protein interactions ( Figure 6), we here report the effects of mutations on its structure ( Figure 9). While mutations were located in the N-terminal region of the molecule, most structural effects were felt downstream in the C-terminal ‘intravirion’ endodomain responsible for interactions with other structural proteins and their recruitment for virion assembly ( Fehr & Perlman, 2015). The mutant make-up of VOC Omicron and *haplotype 15* appear to be major contributors to endodomain distortions, but both maintained the structure of the transmembrane ‘viral envelope’ domain and the hinge region that connects the two domains of the molecule. In sharp contrast, *haplotype 14* twisted the first transmembrane helix. The hinge, which adopts a helix-turn structure inserted into a cavity formed by the triple-helix transmembrane bundle, is a key element for the conformational change and M-protein function, including viral assembly, mediating transition between two conformational states that are in equilibrium ( Zhang *et al*., 2022b). In fact, mutations and deletions in the hinge region are known to inhibit virus formation ( Arndt *et al.*, 2010), explaining the structural conservation of the hinge. Thus, modeling the proteins affected by haplotypes and VOCs confirms the functional centrality of this region. One remarkable finding of these *ab initio* modeling exercises and others we will report elsewhere is that VOC constellations counteracted the more extreme effects of individual haplotypes on protein structure. This strongly suggests that a cooperative activity exists in protein communications that was made explicit in our protein interaction networks. This information will be especially valuable for therapeutic interventions and predictive intelligence applications. They could facilitate understanding of protein reformation, de novo protein and construct design, and formation of molecular complexes.

## Conclusions

Our study uncovers a rationale behind the noisy and dynamic emergence of VOCs and their increasingly complex makeup. While the SARS-CoV-2 genome is evolving in bursts, recombination and recruitment processes gradually generate a number of haplotypes, some of which coalesce into apparently monolithic constellations. Remarkably, seasonal effects decouple these constructs showing they are highly dynamic. Thus, viral evolution appears attuned to the seasonal periodicities of the planet that arise from Earth’s tilted axis of rotation thanks to molecular sensors that probe environmental change and genetically link the structure and function of proteins.

## Data Availability

GISAID EpiCoV™ ( https://gisaid.org): COVID-19 genomic dataset and metadata associated with 11,921,113 sequences available under GISAID identifier EPI_SET_230208zs https://doi.org/10.55876/gis8.230208zs. Access to the data requires registration and agreement to the conditions for use at: https://www.gisaid.org/registration/register/. ModelArchive: AlphaFold2 structural predictions under accession *ma-gca-mprot*: https://dx.doi.org/10.5452/ma-gca-mprot The ModelArchive data are available under the terms of the Creative Commons Attribution-ShareAlike 4.0 International license (CC BY-SA 4.0). bioRxiv: Supplementary Table 2 of Showers *et al*. (posted on March 05, 2021): https://doi.org/10.1101/2021.03.05.433666 The Showers *et al*. data are available under the terms of the Creative Commons Attribution-NoDerivatives 4.0 International license (CC BY-ND 4.0). Zenodo: Seasonal effects decouple SARS-CoV-2 haplotypes worldwide. https://doi.org/10.5281/zenodo.7636393 ( Tomaszewski *et al*., 2023) This project contains:
- The list of 183,276 mutations - The complete list of accession IDs - The GISAID acknowledgement file. Data are available under the terms of the Creative Commons Zero “No rights reserved” data waiver (CC0 1.0 Public domain dedication).

## Software availability

Source code available:

GitHub: Scripts for genome and substitution analysis: https://github.com/gcalab/files/tree/master/F1000Research/scripts

• *Archived source code at time of publication:* https://doi.org/10.5281/zenodo.7636393

License: MIT License

GitHub: Local Colab Fold (version 1.4.0): https://github.com/YoshitakaMo/localcolabfold

• Parameters: --num-recycle 3 --use-gpu-relax

License: MIT License

UCSF Chimera (version 1.16 linux_x86_64): https://www.cgl.ucsf.edu/chimera/download.html

License: Non-commercial license agreement

AS2S server used in CASP (Accessed 2022/12): http://linum.proteinmodel.org/AS2TS/LGA/lga.html

• Superposition parameters: -4 -o2 -gdc -lga_m -stral -d:4.0
• GDT_TS parameters: -3 -o2 -gdc -lga_m -stral -d:4.0 -al

License: Non-commercial license agreement

USAlign Online (2022/12): https://zhanggroup.org/US-align/

License: Non-commercial license agreement

USAlign Offline (Version 20220924): https://zhanggroup.org/US-align/bin/module/USalign.cpp

License: Non-commercial license agreement

TM Score Online (2022/04/15): https://zhanggroup.org/TM-score/

License: Non-commercial license agreement

TM Score Offline (2022/2/27): https://zhanggroup.org/TM-score/TMscore.cpp

License: Non-commercial license agreement
