# Supplementary material for: Seasonal effects decouple SARS-CoV-2 haplotypes worldwide
Source: F1000Res. 2023 Mar 13;12:267. [Version 1] doi: 10.12688/f1000research.131522.1 (PMC10105261; doi:10.12688/f1000research.131522.1)
Supplement: Peer review history

# Peer review: Reviewer response for version 1

**DOI:** 10.5256/f1000research.144373.r168135

**Published:** 2023-04-14

**Authors:** Kyung Mo Kim

**Affiliations:** 1 Division of Polar Life Sciences, Korea Polar Research Institute, Incheon, South Korea

This study aims to investigate the evolutionary history and mechanisms of SARS-CoV-2 viral populations. The authors provide sufficient background information, such as the clinical impact, mutability, evolutionary forces, and infection mechanisms, to help readers understand the remaining parts of the manuscript. By analyzing about 12 million SARS-CoV-2 genomes using phylogenomic and statistical techniques, the authors documented valuable findings in this paper.

 The phylogenetic analysis based on amino acid changes between the genomes showed the emergence of mutants, haplotypes, and VOCs and their evolutionary relationships to each other. Through the prevalence analysis, the authors identified mutants that contribute to the temporal and spatial changes of VOCs, detected decoupling, and newly defined mutant constellations for VOCs. These findings must be valuable for therapeutic interventions against this virus. Additionally, it is worth noting that many VOC mutations appeared during the early phase of the pandemic and that these early-emerging mutations were recruited to form major haplotypes and VOC mutant constellations. The authors further discovered that haplotypes can be unified or diversified by a combination of recruitment, coalescence, decoupling, and loss. Finally, the ab initio modeling produced an interesting result that haplotype divergence resulting in protein structural variations can be balanced by the mutant constellations.

 Overall, this manuscript is well-written, but there are a few minor points to consider. Firstly, it would be helpful to explain the GISAID standards for "high-coverage." Additionally, the authors may provide a rationale for why they only analyzed substitutions with sequence counts greater than or equal to one standard deviation over the mean of the entire sequence set. It might also be worth considering to use the standard deviation of 1.96 that corresponds to a 95% confidence interval. Secondly, the word "accumulation plot" may not be appropriate since a decrease (loss) of mutation prevalence is often observed. I think that “prevalence plot” can be used throughout the text. Finally, the first few sentences in the Molecular Structure section of the Discussion that describe some major strategies for protein structure modeling could be moved to the Introduction or removed from the manuscript.

Is the work clearly and accurately presented and does it cite the current literature?

Yes

If applicable, is the statistical analysis and its interpretation appropriate?

Partly

Are all the source data underlying the results available to ensure full reproducibility?

Yes

Is the study design appropriate and is the work technically sound?

Yes

Are the conclusions drawn adequately supported by the results?

Yes

Are sufficient details of methods and analysis provided to allow replication by others?

Yes

Reviewer Expertise:

Bioinformatics

I confirm that I have read this submission and believe that I have an appropriate level of expertise to confirm that it is of an acceptable scientific standard.

# Peer review: Reviewer response for version 1

**DOI:** 10.5256/f1000research.144373.r168134

**Published:** 2023-04-04

**Authors:** Francisco Solano Muñoz

**Affiliations:** 1 Department of Biochemistry and Molecular Biology, University of Murcia, Murcia, Spain

This is an impressive, long but excellent review about worldwide SARS-CoV-2 evolution. The effect of a number of factors are widely discussed, with nice figures and reasonable classifications. I have spent a long time in reading the article, as I was interested in that and I have learned with the reading. The introduction is nice and clear, containing simple and helpful definitions to follow the manuscript. This is formative and didactic. I encourage the authors to define the term 'clade' in relation to variant and constellations. Another minor point would be at Page 4, concerning the sentence “The first VOCs of SARS-CoV-2 appeared in October 2020, few months after the first wave of the pandemic”. This VOC should be identified and referenced.

 I have no objections but only some questions or curiosities. I recognize that it is a little bit contradictory to report that the review is too long and at the same time to ask for clarifying some points. I hope the authors will be able to conjugate both points.

- This review is in agreement with other studies of the same group, as the Australia´s study, but similar studies from other groups are not discussed. In that way, the review is somehow considered an extension of the Australia study form and endogamic point of view. In that way, for instance, a brief comparison to some recent similar study would be welcome. I propose Cairo *et al.* (2022 ^1^).
- It is stated that only three out of five VOCs, Alpha, Delta and Omicron, became predominant worldwide at some point, completely replacing each other. Beta and gamma VOCs, probably initiated in Brazil and South Africa, have not been considered in this study. Any brief data or discussion about beta and gamma would be welcome. The main reasons why they were not so predominant would be briefly discussed. On the other hand, are they related to some of the 20 haplotypes describes in this review? Do the 20 haplotypes comprise all the worldwide pandemic evolution?
- Please, define viral quasispecies.
- The review dissected the Omicron constellation into 16 haplotypes harboring 78 mutant markers affecting almost half of the SARS-CoV-2 proteome (14 proteins). I wonder why 14. The criteria for choosing 14 proteins and not the whole viral proteome should be described.
- The Discussion is too long indeed. Some paragraphs in the discussion are not related with the title.
- The Title emphasizes the seasonal effects in a so extensive study. I recommend changing the title or reduce the results and discussion.
- Figures 9 and 10 are not directly related to the main objectives of the review. They could be omitted in order to reduce space. I leave this point to the editor and author`s consideration, but the review is too long for other Journals that I have reviewed for. I have no experince in F1000Research.

Is the work clearly and accurately presented and does it cite the current literature?

Partly

If applicable, is the statistical analysis and its interpretation appropriate?

Yes

Are all the source data underlying the results available to ensure full reproducibility?

Yes

Is the study design appropriate and is the work technically sound?

Yes

Are the conclusions drawn adequately supported by the results?

Yes

Are sufficient details of methods and analysis provided to allow replication by others?

Yes

Reviewer Expertise:

Molecular Biology

I confirm that I have read this submission and believe that I have an appropriate level of expertise to confirm that it is of an acceptable scientific standard.

## References

[1] : Worldwide SARS-CoV-2 haplotype distribution in early pandemic. *PLoS One* .2022;17(2) : 10.1371/journal.pone.0263705 e0263705 10.1371/journal.pone.0263705 35171928PMC8849502
